# GRIDSS: sensitive and specific genomic rearrangement detection using positional de Bruijn graph assembly

**DOI:** 10.1101/gr.222109.117

**Published:** 2017-12

**Authors:** Daniel L. Cameron, Jan Schröder, Jocelyn Sietsma Penington, Hongdo Do, Ramyar Molania, Alexander Dobrovic, Terence P. Speed, Anthony T. Papenfuss

**Affiliations:** 1 Bioinformatics Division, Walter and Eliza Hall Institute of Medical Research, Parkville, Victoria, 3052, Australia;; 2 Department of Medical Biology, University of Melbourne, Parkville, Victoria, 3010, Australia;; 3 Department of Computing and Information Systems, The University of Melbourne, Parkville, Victoria, 3010, Australia;; 4 Translational Genomics and Epigenomics Laboratory, Olivia Newton-John Cancer Research Institute, Heidelberg, Victoria, 3084, Australia;; 5 Department of Pathology, University of Melbourne, Parkville, Victoria, 3010, Australia;; 6 School of Cancer Medicine, La Trobe University, Bundoora, Victoria, 3084, Australia;; 7 Department of Medicine, University of Melbourne, Austin Health, Heidelberg, Victoria, 3084, Australia;; 8 Department of Mathematics and Statistics, University of Melbourne, Parkville, Victoria, 3010, Australia;; 9 Peter MacCallum Cancer Centre, Victorian Comprehensive Cancer Centre, Melbourne, 3000, Australia;; 10 Sir Peter MacCallum Department of Oncology, University of Melbourne, Parkville, Victoria, 3010, Australia

## Abstract

The identification of genomic rearrangements with high sensitivity and specificity using massively parallel sequencing remains a major challenge, particularly in precision medicine and cancer research. Here, we describe a new method for detecting rearrangements, GRIDSS (Genome Rearrangement IDentification Software Suite). GRIDSS is a multithreaded structural variant (SV) caller that performs efficient genome-wide break-end assembly prior to variant calling using a novel positional de Bruijn graph-based assembler. By combining assembly, split read, and read pair evidence using a probabilistic scoring, GRIDSS achieves high sensitivity and specificity on simulated, cell line, and patient tumor data, recently winning SV subchallenge #5 of the ICGC-TCGA DREAM8.5 Somatic Mutation Calling Challenge. On human cell line data, GRIDSS halves the false discovery rate compared to other recent methods while matching or exceeding their sensitivity. GRIDSS identifies nontemplate sequence insertions, microhomologies, and large imperfect homologies, estimates a quality score for each breakpoint, stratifies calls into high or low confidence, and supports multisample analysis.

Structural variants (SVs) play a significant role in the development of cancer and other diseases. While significant progress has been made on detection of SVs, they remain less well studied than single nucleotide variation, in part due to challenges in their reliable identification from short-read sequencing data.

Many methods exist to identify SVs using high-throughput sequencing data. These all use one or more of three forms of evidence: read depth, split reads, and discordantly aligned read pairs. Changes in read depth (RD) are associated with copy number variants and imply genomic rearrangements, but when using RD alone, genomic fusion partners cannot be resolved and breakpoint positions are imprecise. Rather, resolution is dependent on the overall sequencing depth and the selected window size used in the analysis. Using paired-end sequencing, clusters of discordantly aligned read pairs (DPs)—i.e., read pairs that align with unexpected orientation or separation, or to different chromosomes—can be used to infer the presence of a breakpoint. Since these are in the unsequenced part of the DNA fragments, DP methods do not identify exact breakpoint locations. Single nucleotide resolution of SVs is important for predicting possible fusion gene products or the impact of a promoter translocation, identifying disrupted tumor suppressors, determining the DNA repair mechanism responsible for the SV, and investigating motifs associated with the breakpoint. This is obtained using split reads (SR), where the sequenced reads span the breakpoint. SR methods find breakpoints by identifying split alignments in which part of the read aligns to either side of a genomic rearrangement, either through direct split read mapping by read aligner, realignment of soft clipped (SC) bases (unaligned bases in partially mapped reads), or split alignment of the unmapped (UM) read in one-ended anchored (OEA) read pairs (read pairs with only one read mapped) (Kidd et al. 2008). These three forms of evidence can be combined in different ways; for example, DELLY combines DP and SR evidence (Rausch et al. 2012), while LUMPY (Layer et al. 2014) uses all three types.

To improve SV calls, short read assembly has also been incorporated into methods in a variety of ways. Assembly of reads obtained from clusters of SC reads (e.g., CREST [Wang et al. 2011]) or OEA read pairs (e.g., NovelSeq [Hajirasouliha et al. 2010]) has been used to form break-end contigs, which extend out and span the breakpoint from each side. In contrast, breakpoint contigs are generated by local assembly of all reads supporting a rearrangement, generating a single contig supporting the variant. Some methods apply targeted assembly to validate the breakpoint calls (e.g., Manta [Chen et al. 2016], SVMerge [Wong et al. 2010], TIGRA [Chen et al. 2014]). Windowed breakpoint assembly has been used (e.g., SOAPindel [Li et al. 2013], DISCOVAR [Weisenfeld et al. 2014]), but detection is limited to events smaller than the window size. Whole-genome de novo assembly has also been used for variant calling (e.g., Cortex [Iqbal et al. 2012]), but its use has been limited, in part due to the computational expense compared to alignment-based approaches.

Here, we present a novel approach to predicting genomic rearrangements from DNA sequencing data, GRIDSS (Genome Rearrangement IDentification Software Suite), which provides multithreaded variant calling from a combination of assembly, split read, and read pair support. The philosophy underpinning GRIDSS is to maximize sensitivity and prioritize calls into high or low confidence, thereby maintaining specificity in the high-confidence call set. To achieve this, we take a three-step approach. First, we filter out reads that align properly; i.e., we extract all reads that might provide any evidence for underlying genomic rearrangements. Second, we perform assembly of all remaining reads using a novel algorithm that utilizes information from the alignment to constrain the assembly. We term this genome-wide break-end assembly, as each contig corresponds to a break-end and only after assembly is the underlying breakpoint and partner break-end identified. Unlike existing break-end assemblers that perform targeted assembly of soft clipped or one-end anchored reads, our approach performs genome-wide assembly of all SC, SR, DP, OEA and indel-containing reads. Similar to split read identification from soft clipped reads, breakpoints are identified by realignment of break-end contigs. Finally, we apply a probabilistic model that combines break-end contigs from each side of the rearrangement with SR and DP evidence to score and call variants.

To perform the genome-wide break-end assembly, we developed a novel assembly approach specifically for the task by extending a positional de Bruijn graph data structure. Originally developed for small indel and base calling error correction of de novo assembly contigs (Ronen et al. 2012), positional de Bruijn graphs add positional information to each node, transforming them into a directed acyclic graph and making use of valuable information generated by the aligner. With appropriate optimization, this is computationally efficient at the genome scale and reduces depth of coverage needed and memory requirements for accurate assembly. To make the best use of data from related samples, sequencing libraries are tracked in the de Bruijn graph using color (Iqbal et al. 2012), and evidence supporting rearrangements is shared between libraries during assembly and variant calling. Since the assembled contigs are longer than the read length, this improves performance in regions of poor mappability. Meaningfully scored variants and a set of useful default filters make GRIDSS easy to use but also a powerful tool for advanced users, who, armed with prior knowledge about expected rearrangements, can identify relevant calls with low support.

## Results

**Figure 1.**
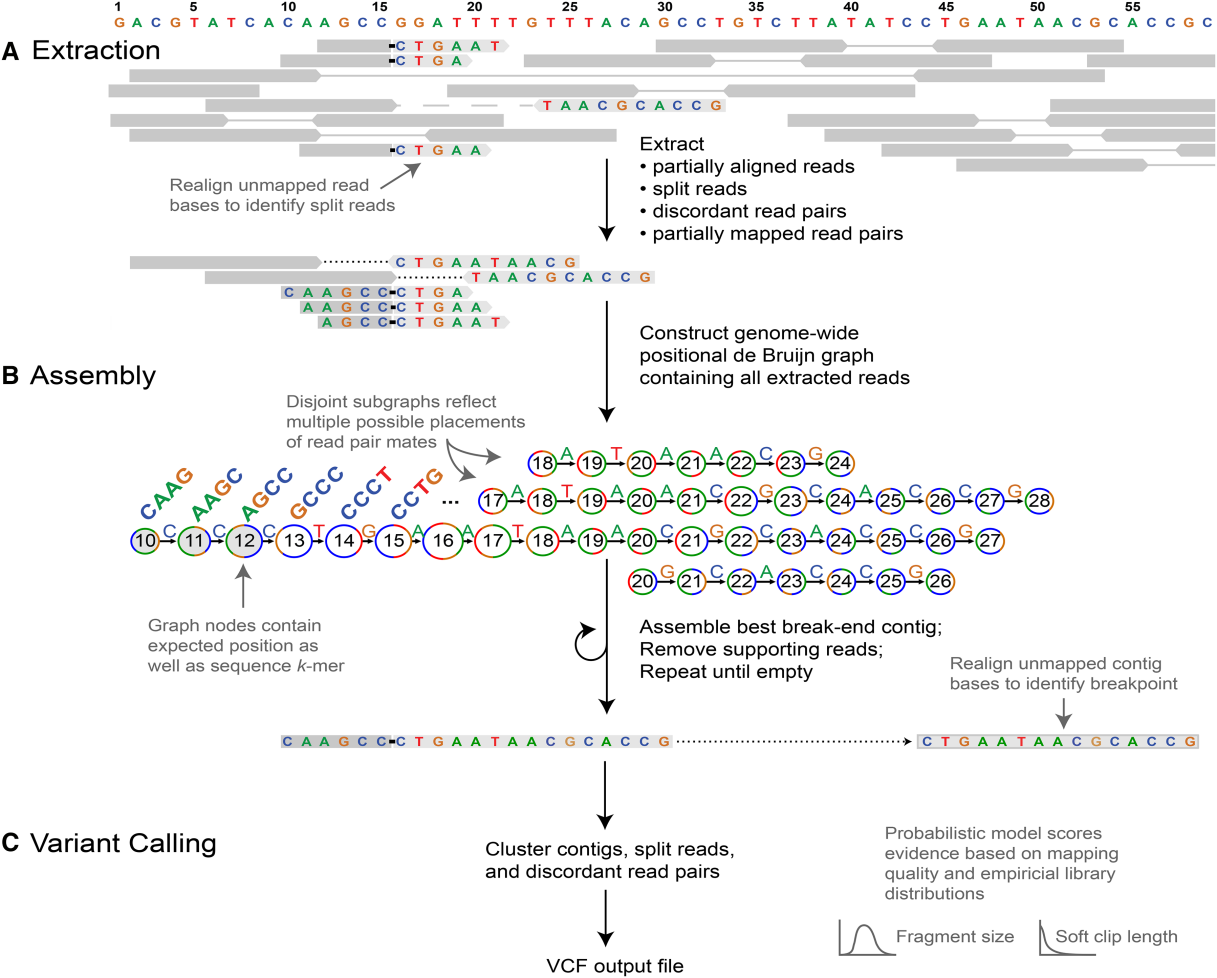
Outline of the GRIDSS pipeline. (*A*) Soft clipped and indel-containing reads as well as discordant and one-ended anchored read pairs are extracted from input BAM files. Split reads are identified through realignment of soft clipped read bases. (*B*) Extracted reads are added to a positional de Bruijn graph in all positions consistent with an anchoring alignment. Break-end contigs are identified by iterative identification of the highest weighted unanchored graph path followed by removal of supporting reads. Unanchored contig bases are aligned to the reference genome to identify all breakpoints spanned by the assembly. (*C*) Variants are called from assembly, split read, and read pair evidence using a probabilistic model to score and prioritize variants.

The GRIDSS pipeline comprises three distinct stages: (a) Extraction; (b) Assembly; and (c) Variant Calling (Fig. 1).

In the Extraction stage, GRIDSS takes as input any number of SAM/BAM alignment files and calculates a number of summary statistics for each sequencing library. SRs are identified by realignment of the unaligned 3′ or 5′ “soft clipped” read bases back to the reference genome. DPs consist of read pairs aligned with inferred fragment sizes shorter or longer than 99.5% of read pairs, in the wrong orientation, or to different chromosomes. All SC, DP, OEA and indel-containing reads are then extracted. At this stage, no breakpoint calling has been undertaken. Reads are scored according to the likelihood of originating from the reference allele based on the read mapping quality, the empirical distributions of the read alignment and library fragment size, and read mapping rate. Extracted reads are passed to the assembly stage, with split reads and discordant read pairs also passed to the variant calling stage.

In the assembly stage, reads are decomposed into a sequence *k*-mer of *k* consecutive bases. These *k*-mers and their genomic locations are incorporated into a positional de Bruijn graph. *K*-mers are considered anchored if the originating read aligns to the reference for all *k* bases and these anchoring *k*-mers are used to constrain the positions in which a read can be assembled. Unanchored *k*-mers from soft clipped reads are placed as if the read were fully mapped. Split and indel-containing reads are treated as two independent soft clipped reads—one for each alignment location. OEA read *k*-mers are placed at all positions compatible with the alignment of the mapped read and the DNA fragment size distribution, with DPs treated as two independent OEA read pairs. Each read *k*-mer is weighted according to the constituent base quality scores and variant support score, with graph nodes weighted by the cumulative supporting weights. Error correction is performed to remove spurious paths caused by sequencing errors. Break-end contigs are iteratively identified by finding the highest weighted unanchored path and extending into anchoring *k*-mers if present. To ensure each read supports only a single contig, reads supporting each break-end contig are removed from the graph when the contig is assembled. Unaligned contig bases are iteratively aligned to the reference genome to identify all genomic rearrangements spanned by the assembly. Data streaming and graph compression is used extensively to keep the assembly memory footprint below 2 GB per thread.

Variant calling occurs in the final stage of GRIDSS. Variants are identified from the overlap in predicted breakpoint positions of assemblies, SRs, and DPs. After identifying and scoring all overlapping support sets, each SR, DP, and assembly is then assigned to the highest scoring variant it supports. High-scoring variants with assembly support from both break-ends are considered high-confidence calls. Nontemplate sequence insertions as well as exact microhomologies and large imperfect homologies are automatically identified in the variant calls.

### Performance on simulated data

To assess the performance of GRIDSS, we simulated heterozygous structural variants with a range of event types (deletion, insertion, inversion, tandem duplication, translocation) and sizes (1 base pair [bp] to 65 kilobase pairs) on human Chromosome 12 (hg19). We compared the GRIDSS results to eight other tools (BreakDancer [Chen et al. 2009], Pindel [Ye et al. 2009], DELLY [Rausch et al. 2012], Hydra-Multi [Lindberg et al. 2015], LUMPY [Layer et al. 2014], Socrates [Schröder et al. 2014], Cortex [Iqbal et al. 2012], and Manta [Chen et al. 2016]) (Supplemental Figs. S1, S2; see Supplemental Material for details).

**Figure 2.**
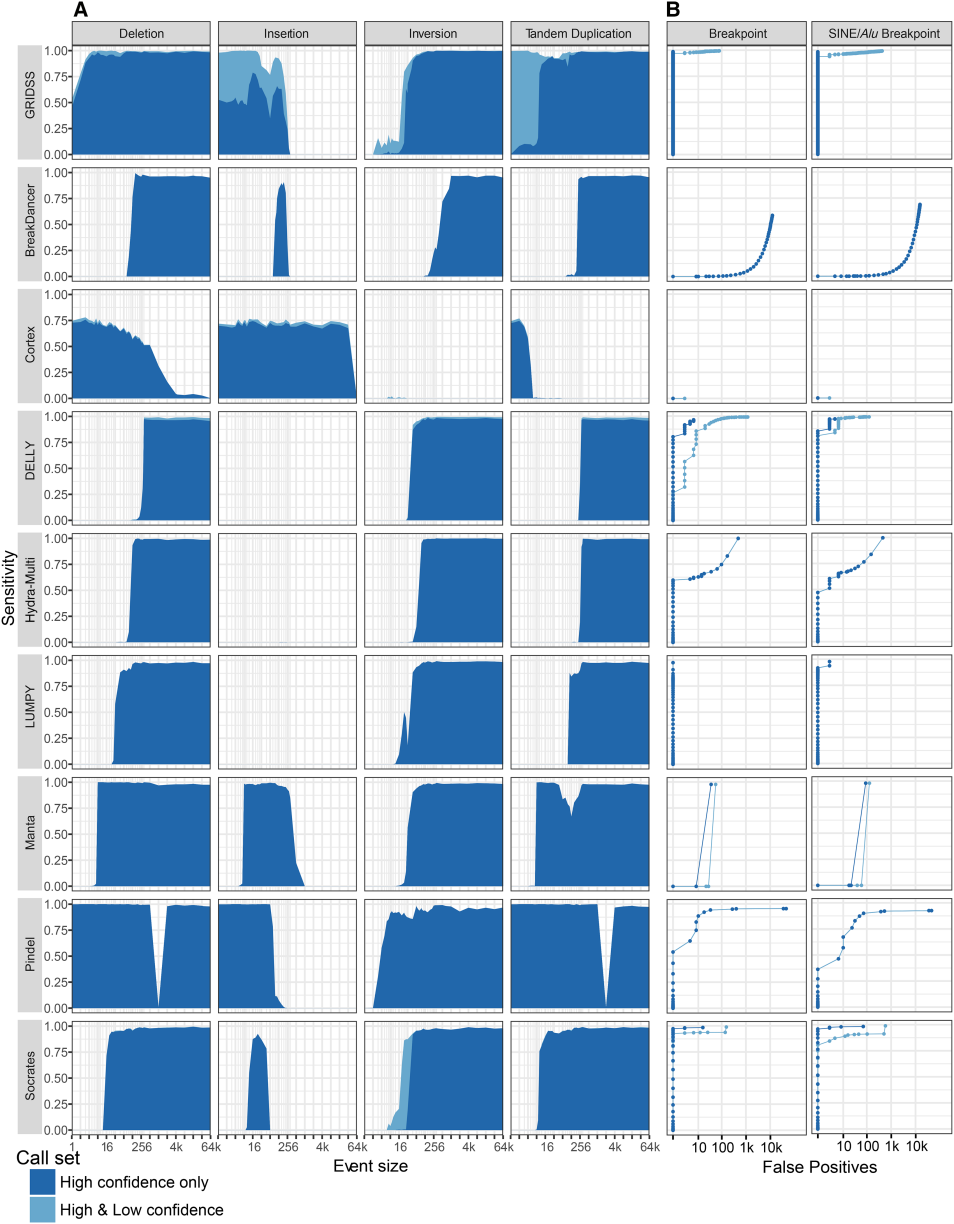
Variant caller performance on simulated heterozygous genomic rearrangements. Different classes of genomic rearrangement were randomly generated against human Chr 12 (hg19), and 60× coverage of 2×100-bp sequencing data was simulated. (*A*) The sensitivity of each method (rows) for each event type (columns) is plotted against event size. (*B*) Receiver operating characteristic (ROC) curves for all breakpoints (*left*) and breakpoints located in SINE/*Alu*s (*right*).

For parameters typical of tumor genome sequencing (60× coverage of 100-bp paired-end reads with a mean fragment size of 300 bp), GRIDSS obtained near-perfect sensitivity across the widest range of event types and sizes (Fig. 2), albeit with Pindel having greater sensitivity on small (<50-bp) events and only the de novo assembly-based caller Cortex able to detect large insertions. For both random breakpoints and breakpoints in SINE/*Alu* elements, GRIDSS obtained the highest *F*-scores (Supplemental Table S1). BreakDancer, DELLY, and Pindel all incorrectly classified breakpoint events as inversion events. When considering caller-reported microhomologies, Cortex, Manta, and GRIDSS identified breakpoints exactly (Supplemental Fig. S14). Except for the detection of insertion events using Cortex, GRIDSS obtained a higher precision than other callers with comparable sensitivity (Supplemental Fig. S2). Analysis against hg38 does not negatively impact GRIDSS performance.

To explore the impact of read length (36–250 bp), DNA fragment size (100–500 bp), sequencing depth (4–100×), and aligner (BWA, NovoAlign, Bowtie 2), GRIDSS was applied to a comprehensive simulation (see Supplemental Material for further details). For reads 50 bp or longer, GRIDSS is able to reliably call and assemble heterozygous genomic fusions at 30× coverage regardless of aligner or fragment size, although some libraries (such as 100-bp paired-end reads with 500-bp fragment size) require as little as 8× coverage. Overall, the *F*_1_-scores of GRIDSS show improved call quality for increasing read length, read depth, and fragment size (Supplemental Figs. S3, S4). For reads 50 bp and shorter, long fragment sizes result in fragmented assembly when using the *k*-mer of 25 (Supplemental Fig. S6). While the precision of calls supported by single-sided or no assembly decreased with coverage as expected, precision of calls supported by reciprocal breakpoint assembly remained near 100% regardless of sequencing depth, read length, library fragment size, or aligner (Supplemental Fig. S5). This demonstrates that requiring reciprocal break-end assembly support, as used by GRIDSS and some other callers (e.g., Wang et al. 2011), is a simple yet powerful false-positive filter. Although frequently overlooked or uncontrolled in experimental designs, our simulation results confirm the significant impact library fragment size has on structural variant calling. Unlike single nucleotide and indel calling, which are relatively independent of library fragment size, the impact of library fragment size on structural variant calling can be the equivalent of up to a twofold change in coverage.

### Performance on cell line data

We next tested GRIDSS on several real sequencing data sets. First, GRIDSS was applied to short-read sequencing data from the NA12878 Illumina Platinum Genomes cell line (50× coverage PCR-free 2×100 bp, accession ERA172924), along with several other structural variant callers. Callers were evaluated against both curated validated call sets (Mills et al. 2011; Layer et al. 2014) and against PacBio and Illumina TruSeq Synthetic Long-Read technology (Moleculo) (Sudmant et al. 2015). As previously (Mills et al. 2011), only deletions longer than 50 bp were considered. So as to not unfairly penalize imprecise callers such as BreakDancer, calls were considered true positives if the breakpoint position error was less than the library fragment size and the event length differed by at most 50% from the validated call set. For the long reads, variant calls required at least three split reads (with each split alignment mapping at least 25% of the long read), or seven reads containing a corresponding indel, to support the event. ROC curves for other callers were obtained by varying the required number of supporting reads as reported by the caller.

**Figure 3.**
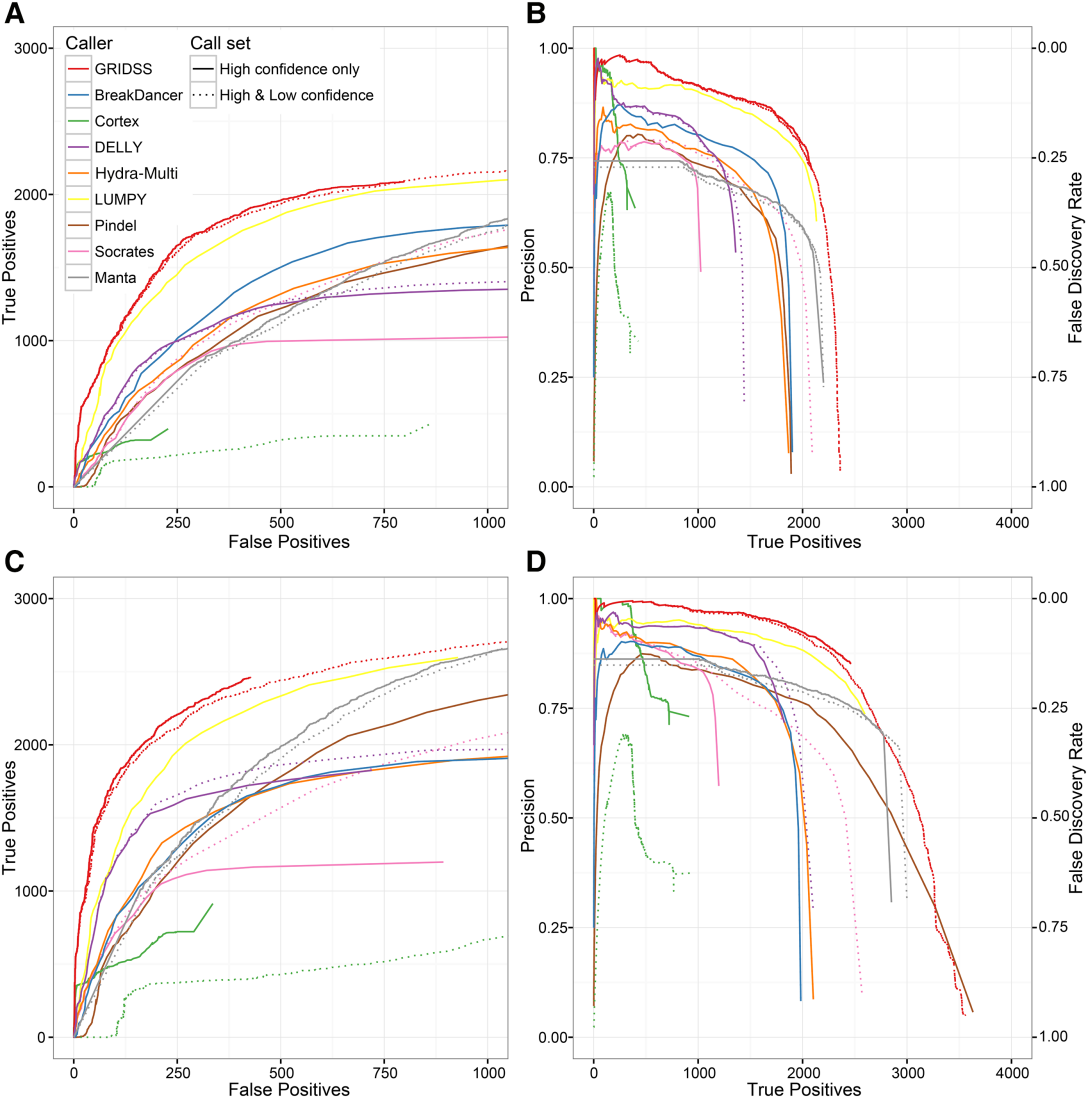
Performance of different SV callers on deletion detection in NA12878 at 50× coverage. Multiple variant calls were compared to both the Mills et al. (2011) validation call set (*A*,*B*) and PacBio/Illumina Tru-Seq Synthetic Long-Read (Moleculo) orthogonal validation data (*C*,*D*). Plots show the number of true positives versus false positives (*A*,*C*) and the precision versus true positives (*B*,*D*). Long-read validation required three split, or seven spanning long reads supporting the call.

For both validated call sets, GRIDSS exhibits considerably better performance characteristics than other callers (Fig. 3). When compared to the Mills et al. (2011) call set, GRIDSS was able to identify the first 1000 true positives with a false discovery rate of 7% (3% using long-read validation data), compared to the next closest method, LUMPY, at 11% (7%).

**Figure 4.**
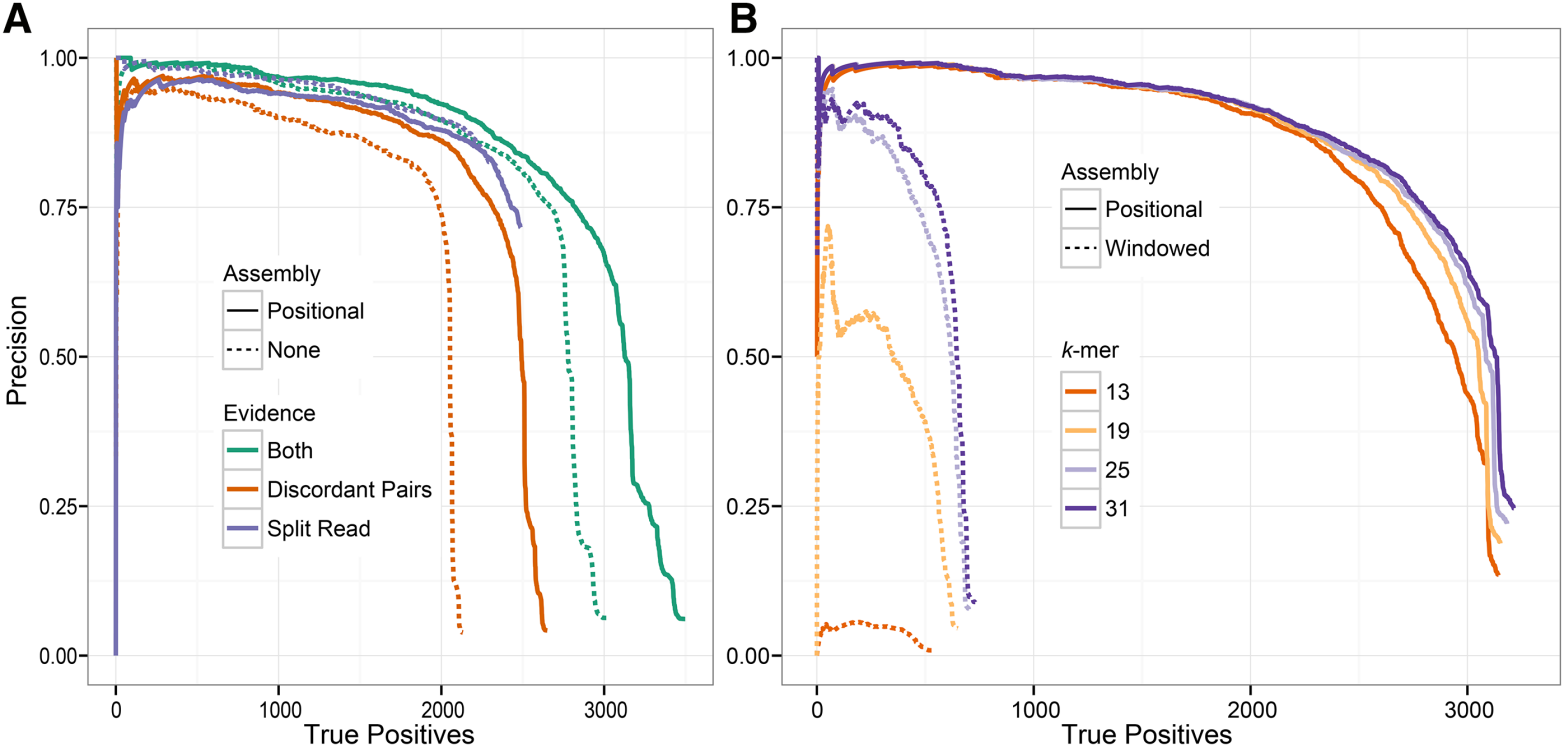
Performance of GRIDSS variant calling and assembly on NA12878 deletions events using long-read orthogonal validation data. Precision versus the number of true positives for different types of support (*A*) and for different *k*-mer sizes (*B*). Assembly of both split reads and read pairs improves both sensitivity and specificity to levels not achievable by either evidence source. Scoring only assembly-supported variants and varying the type of assembly and *k*-mer size demonstrates that robust small *k*-mer break-end assembly can be achieved with positional de Bruijn graph assembly but not windowed de Bruijn assembly.

To determine the relative contributions of split read, read pair, and assembly support, GRIDSS was run on read pair and split read subsets with and without assembly. As expected, using more of the available evidence results in better variant calls (Fig. 4). Assembly improves variant calling for DP and DP+SR evidence but does not improve SR alone, as the assembly contig lengths are limited to the length of the longest soft clip.

To understand the contribution of the positional de Bruijn graph assembly, we tested a windowed break-end assembler using a traditional de Bruijn graph (see the Windowed de Bruijn graph assembly section of the Supplemental Methods) in the GRIDSS framework. By restricting variant scoring to consider only assembly-based support, we compared windowed and positional assembly across a range of *k*-mer sizes against the long-read orthogonal validation data set. Positional de Bruijn graph assembly exhibited a minimal drop in performance from *k*-mers 31 bp to 22 bp, while windowed assembly performance was highly sensitive to *k*-mer size, and sensitivity and specificity were well below those of the positional assembly for all *k*-mers tested (Fig. 4). The poor performance of windowed assembly was traced to the incorrect assembly of *k*-mers occurring in multiple positions within the assembly window (Supplemental Figs. S9, S10). As expected, this phenomenon was especially pronounced in simple repeats and regions of low complexity (Supplemental Figs. S7, S8). As this mode of misassembly does not occur in positional de Bruijn graph assembly, GRIDSS is able to perform assembly with shorter *k*-mers and therefore at lower coverage than either windowed or de novo assembly.

### Application to complex genomic rearrangements

To evaluate the performance of GRIDSS on complex genomic rearrangements, SVs were predicted in three published cancer-associated neochromosome data sets (accession ERP004006) (Garsed et al. 2014; see Supplemental Materials for further details). Each neochromosome contains hundreds of genomic rearrangements identified from the integration of copy number and discordantly aligned read pairs, followed by extensive manual curation. A high concordance with the 1010 curated SVs was obtained, with GRIDSS detecting 98% of the curated calls, 92% with high confidence (Supplemental Fig. S12). GRIDSS obtained a higher concordance with the previously published curated results than other tested methods (BreakDancer, Cortex, DELLY, LUMPY, Hydra-Multi, Pindel, and Socrates) (Supplemental Table S2).

GRIDSS also refined the original call set in three ways. First, GRIDSS calls were made to single-nucleotide resolution. Second, GRIDSS was able to identify 414 additional high-confidence breakpoints; the majority (66%) of these were missing from the curated call set because they were supported by fewer read pairs than the fixed threshold applied in the original analysis or were within 1000 bp of another SV (potentially an issue due to the use of DP evidence alone). Finally, in 5% (64) of the SVs, GRIDSS was able to refine events classified as simple genomic fusions between two locations (A and B) that were in fact compound genomic fusions (from locations A to C to B), where the fragment C was short. In these events, GRIDSS was able to assemble a breakpoint contig at A, fully spanning the C fragment, with the remainder of the contig unambiguously aligning to B. A further 31 compound genomic fusions were identified in which the spanned fragment could not be unambiguously placed. While pure split read methods should also detect these compound rearrangements, the order in which DP and other evidence is applied and how it is applied will impact whether other methods can detect these features. This further refines the picture of complex rearrangements in neochromosomes and provides single-nucleotide resolution of DNA breaks.

### Application to cancer samples

The ICGC-TCGA DREAM8.5 Somatic Mutation Calling Challenge was an international effort to improve methods for identifying cancer-associated mutations and rearrangements in whole-genome sequencing (WGS) data (Boutros et al. 2014). GRIDSS competed in and won subchallenge #5 (https://www.synapse.org/#!Synapse:syn312572/wiki/61498). GRIDSS also performed well as a late entry in historical subchallenge #4, where it was among the best performing tools and detected the highest number of true positive SVs (2950 of 3336 SVs, 0.8843 sensitivity, 0.9749 precision).

To demonstrate the clinical utility of the low false-discovery rate of the GRIDSS high-confidence call set, GRIDSS was used to identify patient-specific DNA biomarkers from tumor biopsies for monitoring of cell-free DNA (Do et al. 2016). Somatic rearrangements were predicted from 40× coverage WGS of primary lung cancers from two patients without matched germline data (see Supplemental Materials for details). Primers were designed for eight candidate SVs from the GRIDSS high-confidence call set (four from each patient), and all eight SVs were validated by real-time PCR (only six were somatic, the remaining two were found to be real germline SVs).

Next, GRIDSS was compared to results from two previously published cancer data sets. First, SVs were identified from sequencing data from a melanoma metastasis and matched germline samples (60× coverage tumor; 30× coverage normal) (Schröder et al. 2014). GRIDSS detected 492 SVs with high confidence and 1,050,525 with low confidence. Of these, 851,981 were supported by three or fewer reads/read pairs. Of the eight somatic events previously predicted by Socrates and validated by PCR (Schröder et al. 2014), all eight (100%) were identified by GRIDSS with high confidence.

Finally, GRIDSS was run on DNA-seq data from the HCC1395 breast cancer cell line and results compared to the published genomic breakpoints, which were predicted to be associated with “validated” fusion genes (Zhang et al. 2016). GRIDSS showed strong concordance with the published results (Supplemental Table S3). Using a 21× coverage subset of the HCC1395 WGS data (only 21× of the 63× is publicly available), GRIDSS identified 23 of the 26 published genomic breakpoints (22 with high confidence, one with low confidence) and failed to identify one breakpoint (manual inspection showed no supporting reads were present in the available data). The remaining two calls not identified by GRIDSS appear to be false positives caused by read-through transcription (see Supplemental Materials for details). Additionally, GRIDSS identified genomic breakpoints associated with a further three of the validated fusion genes.

### Application to *Plasmodium falciparum* genome data

**Figure 5.**
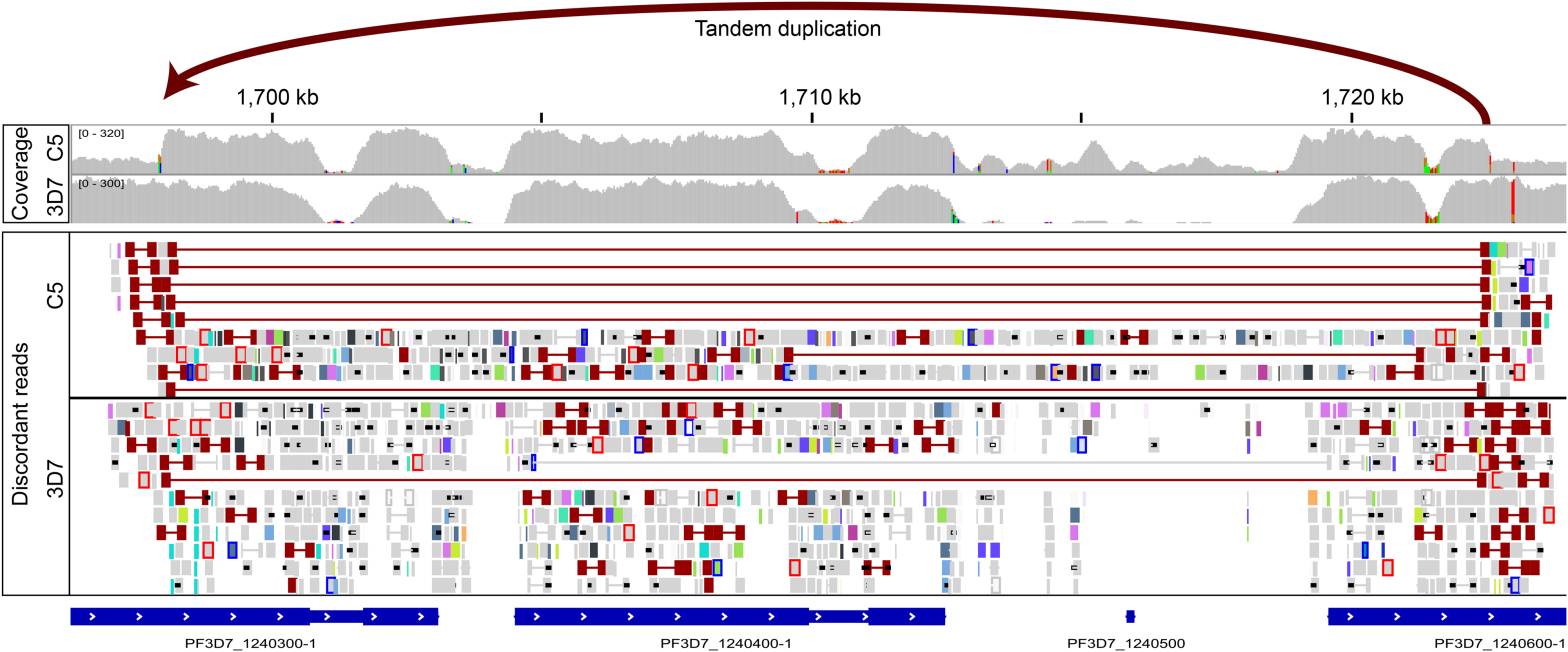
A tandem duplication identified in a var gene region of the AT-rich *Plasmodium falciparum*. Coverage is shown for two samples of *P. falciparum*—a genetically modified line (C5), which was derived from the parental laboratory strain (3D7). The AT-rich genome shows high coverage in genes, which drops to very low levels in the AT-rich nonexonic regions. A change in copy number is apparent in the C5 coverage. GRIDSS detected the underlying tandem duplication in the C5 vaccine candidate (indicated). The supporting discordant read pair (DP) evidence is shown for both strains. Weak evidence (one read pair) for this rearrangement was also detected in the parental population, indicating that the SV was subclonal in this population. This evidence contributed to the positional de Bruijn graph assembly.

To test the behavior of GRIDSS on a challenging AT-rich nonmammalian genome, it was applied to a laboratory strain of *Plasmodium falciparum* that was genetically modified for use as a live, attenuated malaria vaccine (C5) and to the parental 3D7 population (accession PRJE12838). In the vaccine candidate, a *Plasmodium* gene *KAHRP* was knocked out by insertion of a construct containing a processed copy of the human *DHFR* transcript. In addition to identifying the insertion of the construct into the *KAHRP* locus, GRIDSS detected eight of the nine exon-splicing events associated with the processed *DHFR* transcript with high confidence, while the ninth splicing event was found in the low-confidence call set. This demonstrates the value of GRIDSS’ sensitivity and prioritization of calls. Finally, a tandem duplication was also identified by GRIDSS in one of the var gene regions and supported by a copy number change (Fig. 5). Var genes are a large and complex gene family, and the var gene regions are prone to recombination. The duplication was clonal in the C5 candidate and present at low frequency in the parental population (supported by one read), which was utilized in the positional de Bruijn graph to identify the rearrangement.

## Discussion

We have developed the GRIDSS software package, which performs genome-wide break-end assembly and combines assembly, split read, and discordant read pair evidence using a probabilistic model to identify and score structural variants. It automatically detects nontemplate sequence insertions and both exact microhomologies and large imperfect homologies. Through comparison with existing split read, read pair, assembly-as-validation, and de novo assembly approaches on both simulated and real data, we have shown that our approach significantly improves both sensitivity and specificity of variant calling and that this improvement is achieved by performing break-end assembly of SR, DP, OEA, and SC reads prior to variant calling. We also demonstrated that GRIDSS is effective on real data from human tumors as well as nonmammalian organisms.

GRIDSS is designed to make the most of available evidence. The break-end assembly algorithm is able to make use of SR, DP, OEA, SC, and indel-containing reads and utilizes information generated by the aligner via a positional de Bruijn graph. By combining SR, DP, and assemblies into a unified probabilistic model, GRIDSS is able to call variants even in the absence of any two of these signals. Such an approach is a distinct advantage over callers such as DELLY that require a threshold signal strength in one signal before considering any others. Fundamentally, GRIDSS detects SV breakpoints and has been designed to handle complex rearrangement scenarios where assumptions regarding ploidy cannot be made. Both genotyping and incorporation of read depth is left to downstream programs such as SVTyper (Chiang et al. 2015) and CONSERTING (Chen et al. 2015).

GRIDSS is designed to be highly sensitive and not miss any putative genomic rearrangements. GRIDSS uses variant score and the presence of assembly support from both sides of the rearrangement to classify variants as high- or low-confidence. The high-confidence set provides an immediately usable call set with high specificity, which is particularly important in clinical applications. The retention of low-confidence calls enables analysis requiring high sensitivity.

Our novel genome-wide break-end assembly approach is made possible through the utilization of a positional de Bruijn graph to incorporate read alignment constraints into the graph structure itself. This allows us to perform genome-wide assembly without targeting or windowing and use a *k*-mer size half that required for de novo de Bruijn graph assembly. This small *k*-mer in turn results in improved assembly at lower levels of coverage. Although our approach results in a graph two orders of magnitude larger than the equivalent de novo assembly de Bruijn graph, we are able to perform genome-wide break-end assembly faster than a number of existing targeted breakpoint assembly implementations through graph compression, data streaming, multithreading, reference-supporting read exclusion, and extensive use of dynamic programming and memoization. By demonstrating that GRIDSS performance is comparable to existing callers when only discordant read pairs or split reads are considered, we show that it is the novel incorporation of positional de Bruijn graph-based whole-genome break-end assembly into the variant calling process that is the key to the superior performance of GRIDSS.

## Methods

GRIDSS has been designed for 36- to 300-bp Illumina paired-end or single-end DNA sequencing data and accepts any number of coordinate sorted SAM, BAM, or CRAM input files, with no requirement for matching read lengths between input files. Mate-pair libraries are currently not supported. User-supplied categories for each input file allow for somatic and multisample calling with support and variant scoring broken down per category. Variant calls are output to VCF using the break-end notation (VCFv4.2).

### Extraction of supporting reads

An initial parse of the read alignments is performed to collect library metrics. Metrics are calculated independently for each input file, thus allowing libraries from multiple related samples (or unrelated samples from a population) to be processed together. The following metrics are gathered in the initial pass for each input file: read alignment CIGAR element length distribution, fragment size distribution, and counts of total reads, mapped reads, unmapped reads, total read pairs, read pairs with both reads unmapped, read pairs with one read unmapped, read pairs with both reads mapped, maximum read length, and maximum mapped read length. The fragment size distribution is calculated using Picard (http://broadinstitute.github.io/picard) CollectInsertSizeMetrics.

Next, reads partially aligning to the reference (reads containing indels or soft clipped bases) and read pairs in which the inferred fragment size falls outside the expected fragment size distribution (default shorter or longer than 99.5% of read pairs) of the library, with incorrect orientation, or with only one read mapped are extracted to intermediate files. GRIDSS does not utilize read pairs in which both reads are unmapped. To reduce false positives, a number of optional filters are applied to the extracted reads (see Supplemental Material for details).

### Identification of split reads

Split reads are identified by aligning the soft clipped bases of partially aligned reads back to the reference using BWA (default) or another aligner that is compliant with the SAM file format specifications (such as Bowtie 2). Reads for which the soft clipped bases are uniquely aligned to the reference (default MAPQ ≥10) are considered to provide split read support. Reads containing insertions or deletions in the read alignment are treated as split reads aligning to either side of the indel.

### Positional de Bruijn graph assembly

A positional de Bruijn graph is a graph where each node contains a sequence of *k* bases and the position at which those *k* bases are expected to occur. The positional de Bruijn graph *G* = (*V*, *E*) consists of the vertex set *V* and the edge set *E*⊆ *V* × *V*. Let (*S*_*n*_, *p*_*n*_) ∈ *V* be the tuple *n* where *S*_*n*_ = (*b*_*n*,1_, *b*_*n*,2_, …, *b*_*n*,*k*_) is a *k*-mer of *k* bases, and *p*_*n*_ ∈ ℤ is the expected genomic position of the *k*-mer. By definition, nodes *x*, *y* ∈ *V* are connected if they have adjacent *k*-mers and positions:

$$(x,y)\in E\equiv p_{x}+1=p_{y}\wedge \forall_{i=1}^{k-1}b_{x,i}=b_{y,i+1}.$$

Unlike a traditional de Bruijn graph, *G* is a directed acyclic graph since $\forall_{i,j}(n_{i},n_{j})\in E\to p_{i}<p_{j}$; any cycle would contain an edge in which *p*_*i*_ ≥ *p*_*j*_. That is, all paths through the graph are simple (not self-intersecting) paths since traversal of every edge must advance the position by a single base. This allows algorithms such as the longest path problem that would require exponential time on a de Bruijn graph to be completed in polynomial time when applied to a positional de Bruijn graph. In addition to the *k*-mer and position, the following node attributes are used:

- *w*(*n*) is the node weight corresponding to the Phred-scaled probability of the supporting read *k*-mers. The probability of the *i*th *k*-mer of read *r* is given as the joint probability of every *k*-mer base being correctly called by the sequencer and the aligner mapping the read to the correct location. That is,
  $$\;w(r,i)=-10\log_{10}\left(1-\left(1-10^{\frac{-mapq(r)}{10}}\right)\cdot \overset{i+k}{\underset{\;j=i}{\prod }}\left(1-10^{\frac{-baseq_{j}(r)}{10}}\right)\right),$$
  where *baseq*_*j*_(*r*) is the Phred-scale base quality score of the *j*th base, and *mapq*(*r*) is the mapping quality score of read *r*. As w(n)=∑w(r,i).
- *anchored*(*n*) is a Boolean flag indicating whether the node bases are fully aligned to the reference genome in any supporting read. Note that this definition does not require the read bases to actually match the reference and both matched and mismatched base alignments are considered to be anchored if aligned.
- *support*(*n*) is the set of reads providing support for the given *k*-mer at the given position.

### Assembly graph construction

All extracted reads are added to one of two positional de Bruijn graphs based on the expected break-end orientation. Reads supporting a rearrangement after the anchored position are added to the forward graph, whereas reads supporting a rearrangement before the anchored positions are added to the backward graph. Each read *k*-mer is added to the graph at all expected mapping positions. For a soft clipped read, each read *k*-mer is added to *G* at the position the *k*-mer would start at if the entire read were mapped to the reference. Split reads are treated as two independent soft clipped reads. For read pairs, read *k*-mers are added at all positions in which the read pair would be considered concordantly mapped based on the mapping location of the partner. For discordant read pairs, each read is added based on the anchoring location of the mate irrespective of the actual mapping location of the read, whereas one-end anchors are only added at one location, as the unmapped read can provide no positional constraints on its partner.

Using this method of graph construction, correctly assembled unanchored paths are limited in length to less than the read length if supported by only soft clipped or split read evidence and by the maximum concordant fragment size if supported by read pair evidence.

### Assembly graph error correction

Base-calling error correction is performed by collapsing similar paths. Paths are scored according to the sum of the node weights. A path A is collapsed into an alternate path B if both the total path weight of A is less than B, A and B differ by less than a fixed number of bases (default 2), and either both paths are same length and share the same start and end node (bubble popping), or A shares a start or end node with B and contains a single terminal leaf node (leaf collapse). By default, error correction is only performed on paths of length less than twice the read length that are either simple bubbles or terminal leaves.

For each node, all leaf and branch paths under the maximum length originating from the node are identified by traversal of branchless descendants. For each path identified, breadth first graph traversal is performed to identify candidate paths to merge. Memoization is used to track the optimal paths to each node thus reducing worst-case traversal complexity from exponential time to quadratic.

### Break-end contig assembly

A break-end contig path consists of a sequence of adjacent unanchored nodes, optionally flanked by a sequence of anchored nodes. Paths flanked by anchoring nodes are called before unflanked paths. Maximally weighted paths are calculated in the same manner as error correction traversal with breadth-first traversal with memoization of the highest-weighted partial path at each node.

Once the maximally weighted path has been determined, the path is extended into flanking anchored nodes until the anchored path length exceeds both the maximum read length and the unanchored path length. Once the maximally weighted path contig is called, all reads supporting any unanchored *k*-mer on the contig path are removed from the graph. The removal of supporting reads from all graph nodes ensures that each read contributes to a single assembly only. Contigs are iteratively called until no nonreference *k*-mers remain in the graph.

Assembly contigs supported by less than the minimum required support (by default, three reads) and unanchored contigs shorter than the read length are filtered.

### Contig error correction

While positional information at nodes significantly reduces the rate of misassembly compared to windowed assembly, branch traversal introduces new modes of misassembly. When a read pair is self-intersecting or contains repeated *k*-mers, the resultant contig will loop for as long as the fragment size window will allow. This misassembly can occur even with a single read. For example, with *k* = 4, the single read TAAAAC expected to start at one of the positions in the interval [10, 15] will result in the highest weighted path of TAAAAAAAAC starting at position 10. To prevent such misassemblies, *k*-mer chaining of the supporting reads is used to truncate the called path at the first *k*-mer transition not supported by any constituent read. Truncation is performed starting from both the start and the end of the contig with the highest weighted truncated path called, preferentially calling anchored paths. Each supporting read is aligned to the contig position with the greatest number of matching *k*-mers (breaking ties toward the truncation start *k*-mer) and all *k*-mer transitions supporting by the read are marked. Once all supporting reads have been processed, the contig is truncated at the first *k*-mer transition not supported by any reads.

### Contig realignment

Once an assembly contig has been called, a multistage realignment process is used to identify the breakpoint supported by the contig. Assemblies containing at least one anchored base undergo Smith-Waterman (local) realignment around the expected contig position. Assemblies that fully align to the reference are treated as a fully aligned indel-spanning assembly if an indel is present in the alignment or filtered out as a false positive if the full alignment contains no indels. For unanchored assemblies with no soft clip or split read support, a breakpoint position interval is calculated based on the breakpoint interval consistent with the greatest number of supporting read pairs.

The contig bases not anchored to the reference are aligned using an external aligner (by default, Bowtie 2) in local alignment mode using the same alignment thresholds used for identifying split reads. For assembly alignment, if the external aligner identifies the best alignment to be a soft clipped alignment, these soft clipped bases are again aligned, with such recursive alignment limited to a depth of four. This compound realignment results in assembly support not only for the breakpoint site identified by the initial realignment but also for any additional breakpoints spanned by the assembly. This approach allows accurate classification of complex rearrangements such as those present in neochromosomes formed through chromothripsis and the breakage-fusion-bridge process.

### Graph compression

To reduce the graph size, a compressed representation of the positional de Bruijn graph is used. Nodes with a single successor connected to nodes with a single ancestor correspond to a branchless path and are compressed into a single path node. Similarly, nodes with adjacent positions and matching *k*-mers, weights, and reference status are compressed using a position interval. The resultant path nodes are of the form (*start*, *end*, (*kmer*_1_, *weight*_1_, …, *kmer*_*n*_, *weight*_*n*_), *anchored*), representing the set of nodes ${⋃}_{\;p=start}^{end}{⋃}_{i=1}^{n}\left(\;p,kmer_{i},weight_{i},anchored\right)$.

### Streaming assembly

Since all nodes are associated with a genomic position and only have edges to adjacent genomic positions, all graph operations only affect the local subgraph near the genomic position. Exploiting this, positional de Bruijn graph assembly is performed in a single streaming pass over the input reads. Coordinate sorted records are streamed through the following five internal processes, each processing records within a genomic position window size determined by the maximum read length and fragment size:

1. Extraction: Each read is converted into constituent positional de Bruijn graph nodes. To reduce the graph size, positional intervals are stored implicitly with each extracted node defined over a positional interval.
2. Positional aggregation: Overlapping nodes from multiple reads are split into disjoint aggregated nodes and graph edges calculated and cached.
3. Path compression: Nonbranching aggregate node paths are compressed into path nodes.
4. Error correction: Bubble popping and leaf collapse error correction is performed to remove base-calling artifacts.
5. Assembly: Maximal path contig calling is performed such that whenever a maximal path is encountered, the streamed assembly graph loads and traverses all alternate paths which any reads supporting the maximal path could also support. Since each read can contribute to graph node positions over an interval no wider than the concordant fragment size plus the read length, the globally maximal path containing any given read must overlap this interval and is thus a local graph operation. A contig is called whenever the subgraph has loaded all potential alternate paths for the highest weighted maximal path encountered. Since all reads contributing to the called path must have been fully loaded for all such potential alternate paths to have been traversed, all reads contributing to the contig are fully removed from the subgraph immediately. This approach ensures that any contig called is the globally maximal contig containing the given reads.

This assembly algorithm is implemented by memoization of all maximal paths of the streaming subgraph. The starting position of paths for which a potential successor node has not yet been loaded are tracked in a frontier and when the end position of the maximal path plus the concordant fragment size is earlier than both the earliest start position of the frontier path and the start position of the next node to be loaded, the maximal contig is called.

As a full recalculation of all maximal paths in the subgraph after supporting reads have been deleted is unnecessary, only paths affected by node removal are recalculated. For sufficiently high-coverage data, there will be enough concordant fragments with unexpectedly long or short fragment size that a background signal supporting small indels everywhere across the entire genome will be present. When these reads are assembled, this signal results in long unanchored paths up to multiple megabases in size. To suppress this background noise, contigs and paths longer than the maximum expected size are filtered and thresholds are placed on both the size and the genomic width of the loaded subgraph (see Supplemental Materials).

### Probabilistic variant scoring model

To estimate the quality of predicted structural variants, we score variants according to the Phred-scaled probability of originating from the mapped locations without any underlying structural variations. The Phred score *Q* of a probability *P* is given by *Q* = −10log_10_(*P*). Given a read pair or split read *r* mapping to genomic locations *a* and *b*, and the event *M* that the mapping is correct, the score assigned to *r* is given by $\mathrm{Pr}(r)=\mathrm{Pr}(r\cap M)+\mathrm{Pr}(r\cap \bar{M})=\mathrm{Pr}(M)\cdot \mathrm{Pr}(r|M)+\mathrm{Pr}(\bar{M})\cdot \mathrm{Pr}(r|\bar{M})$. The probability of correct mapping Pr(*M*) is determined directly from the Phred-scaled mapping quality scores *mapq*_*a*_(*r*), *mapq*_*b*_(*r*) defining the probability of incorrect read alignment: $\mathrm{Pr}(M)=(1-10^{mapq_{a}(r)/10})(1-10^{mapq_{b}(r)/10})=1-\mathrm{Pr}(\bar{M})$ since Pr(*M*) requires both mapping locations to be correct. In the case of incorrect mapping, *r* is uninformative and $\mathrm{Pr}(r|\bar{M})=1$. In the case of correct mapping, Pr(r|M) is determined empirically from the relevant library distribution.

For split reads, correct mapping with no SV implies the alignment is artifactual. We model the artifactual alignment rate from the empirical soft clip length distribution of the library; thus, $\mathrm{Pr}(r|M)=P_{sc}(l_{sc}(r))$ where *l*_*sc*_(*r*) is the length of the soft clip of *r* before split read identification, and *P*_*sc*_ is the library soft clip length distribution.

For read pairs, correct mapping can be caused either by a chimeric fragment *CF*, or an unexpectedly large/small originating fragment: $\mathrm{Pr}(r|M)=\mathrm{Pr}(r\cap CF|M)+\mathrm{Pr}(r\cap \bar{CF}|M)=\mathrm{Pr}(CF|M)\cdot \mathrm{Pr}(r|M\cap CF)$ $+\mathrm{Pr}(\bar{CF}|M)\cdot \mathrm{Pr}(r|M\cap \bar{CF})$ $\mathrm{Pr}(r|M\cap \bar{CF})$ is given as *P*_*rp*_(*ifs*(*r*)) where *ifs*(*r*) is the fragment size of *r* inferred from the read mapping locations, and *P*_*rp*_ is determined from the library fragment size distribution inferred from all mapped read pairs. Chimeric fragment alignments are considered uninformative and Pr(CF|M) is taken to be *p*_*d*_, the rate of read pair mapping in which the inferred fragment size exceeds more than 10 mean absolute deviations from the median library fragment size. Thus, for read pairs $\mathrm{Pr}(r|M)=p_{d}+(1-p_{d})\cdot P_{rp}(ifs(r))$.

Split reads originating from indels use the corresponding distribution of insertion or deletion alignment operations instead of the soft clip distribution. Assemblies are modeled as a set of constituent reads, with the anchored mapping quality defined as the greatest mapping quality of the constituent reads and unanchored mapping quality determined by the assembly alignment mapping quality. Constituent soft clipped reads and reads with unmapped mates are treated as split reads and discordant read pairs for the purpose of determining assembly quality, with the caveat that reads with unmapped mates use *p*_*u*_ in place of *p*_*d*_. This assembly scoring model improves variant calling by rescuing poorly mapped reads, increasing the score of variants supported by assembly, and promoting SC and UM reads to SR and DP reads within the context of the assembly, thus allowing these reads to be used as input to the variant calling. For computational efficiency, scoring calculations are approximated using the maximum Phred score of the constituent terms.

### Variant calling using maximal cliques

Variants are scored according to the level of support provided by SR, DP, and assembly evidence combined. Supporting evidence can be summarized as the tuple (*s*_*l*_, *e*_*l*_, *s*_*h*_, *e*_*h*_, *d*_*l*_, *d*_*h*_, *w*) where the intervals [*s*_*l*_, *e*_*l*_] and [*s*_*h*_, *e*_*h*_] are the genome intervals between which a breakpoint is supported, *d*_*l*_ and *d*_*h*_, the direction of the supported breakpoint, and *w* the weight of the evidence as defined by the evidence scoring model. Since each piece of supporting evidence is considered to be independent, and evidence scores are expressed as Phred scores, the score for any given variant is equal the sum of the scores of evidence supporting the variant breakpoint.

Calculating the total support weight for all putative breakpoints is equivalent to finding all maximum evidence cliques, that is, all sets of evidence providing consistent support for a breakpoint such that no more evidence can be added and the set remain consistent. Since both direction *d*_*l*_ and *d*_*h*_ must match if evidence is to mutually support a breakpoint, the evidence set can be divided into the four ++, +−, −+, −− directional subsets, which reduces the problem to weighted maximum clique enumeration of a rectangle graph (Lee 1983). Maximal clique enumeration is performed in a single in-order pass over evidence in polynomial time.

Unfortunately, this approach can result in reads providing support to multiple independent breakpoints. Since each read will have originated from, at most, one of the competing explanatory variants, a second pass is made and a greedy assignment is performed in which each piece of evidence is assigned to only the highest scoring variant it supports.

### Detection of microhomology and nontemplate sequence insertions

Depending on the pathway involved in DNA repair resulting in a structural variant, there may exist either sequence homology at the breakpoints or nontemplate sequence inserted during the repair. Microhomology at the breakpoints introduces uncertainty into the breakpoint position call, which is resolved if nontemplate sequence is also present. As a consequence of aligner behavior, unhandled sequence homology can result in two separate variant calls (one at each edge of the homology) for a single event, as aligners are able to map read to the homologous sequence at both sides of the breakpoint. Unhandled nontemplate sequence insertions result in incorrect breakpoint sequence and event size calculation and, for coding variants, result in incorrect gene fusion transcript prediction.

GRIDSS factors in both breakpoint sequence microhomology and nontemplate sequence insertions when performing variant calling. For microhomologies, the breakpoint location for split reads and assemblies is expanded from a single base to an interval of the length of sequence homology between the read/assembly and the reference sequence at either side of the predicted genomic rearrangement. The nominal position of the called breakpoint is considered to be the center of the homology and is reported using the standard HOMSEQ, HOMLEN, and CIPOS fields in the VCF output. Nontemplate sequence insertions are included as an additional component of the variant call in a similar fashion to Socrates (Schröder et al. 2014).

In addition to the standard VCF homology fields, GRIDSS reports the inexact homology in the nonstandard IHOMPOS field. The inexact homology length is calculated for each break-end by performing local Smith-Waterman alignment of the breakpoint sequence to the reference sequence up to 300 bp on either side of the break-end.

Detection of nontemplate sequence insertions is limited by the assembly contig length. Since each read contributing to a break-end assembly requires an anchoring alignment, the maximum contig length is limited by the library fragment size distribution. Microhomology detection requires unambiguous alignment of supporting read pairs spanning the microhomology; thus, the length is similarly limited by the library fragment size distribution.

### Software development methodology

GRIDSS was developed as professional quality software using a test-driven development methodology. To develop new functionality, test cases were first written describing the expected behavior under normal and error conditions. Once such failing test cases have been written, code implementing the feature is written, thus ensuring that the feature is functioning as expected. Bug fixing is performed by first creating a failing test case reproducing the error, then updating the implementation to correct the error. As a result, an extensive test suite composed of over 1200 test cases has been developed. Git is used as a version control system. All tests are rerun prior to each release, ensuring regression faults in existing functionality are not introduced when new features are added.

Bug fixes and enhancement in libraries used by GRIDSS have been contributed back to these upstream libraries. Maven3 (https://maven.apache.org/) is used for build packaging, and a single precompiled binary including all dependencies (except the external aligner) is produced for each release. All parameters used by GRIDSS (including the choice of external aligner used) have been externalized into a configuration file able to be modified by advanced users. Semantic versioning is used for release versioning.

GRIDSS is implemented in Java 1.8. GRIDSS has been designed as a modular software suite. Although an all-in-one entry point is included, each stage of the GRIDSS pipeline, including the break-end assembler, can be run as an independent program or replaced with an equivalent implementation. Example scripts for single sample, somatic, and multisample pipelines are provided. Java utility programs and R (R Core Team 2017) scripts to convert GRIDSS VCF break-end format to more user-friendly formats for downstream filtering and analysis are also provided.

### Software availability

GRIDSS has been released as free and open source software under a GNU General Public License (GPL version 3). Source code is included in the Supplemental Materials. The latest source code and precompiled binaries are available at https://github.com/PapenfussLab/gridss. All scripts required for independent replication of results presented in this paper are available as part of the Supplemental Materials source code and can also be found at https://github.com/PapenfussLab/sv_benchmark.

## Supplementary Material

Supplemental Material
