# Supplementary material for: GRIDSS: sensitive and specific genomic rearrangement detection using positional de Bruijn graph assembly
Source: Genome Res. 2017 Dec;27(12):2050–60. doi: 10.1101/gr.222109.117 (PMC5741059; doi:10.1101/gr.222109.117)
Supplement: Supplemental Material [file supp_27_12_2050__index.html]

GRIDSS: sensitive and specific genomic rearrangement detection using positional de Bruijn graph assembly — Supplemental Material 

# GRIDSS: sensitive and specific genomic rearrangement detection using positional de Bruijn graph assembly

## Supplemental Material

- Supplemental\_Material.docx
- Supplemental\_Fig\_S1.pdf
- Supplemental\_Fig\_S2.pdf
- Supplemental\_Fig\_S3.pdf
- Supplemental\_Fig\_S4.pdf
- Supplemental\_Fig\_S5.pdf
- Supplemental\_Fig\_S6.pdf
- Supplemental\_Fig\_S7.pdf
- Supplemental\_Fig\_S8.pdf
- Supplemental\_Fig\_S9.pdf
- Supplemental\_Fig\_S10.pdf
- Supplemental\_Fig\_S11.pdf
- Supplemental\_Fig\_S12.pdf
- Supplemental\_Fig\_S13.pdf
- Supplemental\_Fig\_S14.pdf
- Supplemental\_Table\_S1.xlsx
- Supplemental\_Table\_S2.xlsx
- Supplemental\_Table\_S3.xlsx
- Supplemental\_gridss\_source.zip
