# Supplementary material for: GRIDSS: sensitive and specific genomic rearrangement detection using positional de Bruijn graph assembly
Source: Genome Res. 2017 Dec;27(12):2050–60. doi: 10.1101/gr.222109.117 (PMC5741059; doi:10.1101/gr.222109.117)
Supplement: Supplemental Material [file supp_gr.222109.117_Supplemental_Material.docx]

**GRIDSS: sensitive and specific genomic rearrangement detection using positional de Bruijn graph assembly**

Daniel L Cameron, Jan Schroeder, Jocelyn Sietsma Penington, Hongdo Do, Ramyar Molania, Alexander Dobrovic, Terence P Speed, Anthony T Papenfuss

**SUPPLEMENTAL MATERIAL**

TABLE OF CONTENTS

SUPPLEMENTAL RESULTS 2

Simulated data 2

Contig assembly rate 6

Subgraph Assembly 6

Assembly comparison results 7

Performance in repetitive regions 9

Performance on complex somatic rearrangements 10

Comparison to other callers on neochromosome call set 11

Validation on lung cancer 11

HCC1395 cell line genomic breakpoints identification 13

Variant scoring model comparison 14

Runtime Performance 15

Assembly Memory Consumption Breakdown 16

SUPPLEMENTAL METHODS 17

Read filtering 17

Coverage blacklist 19

Breakpoint position error margin 19

Windowed de Bruijn graph assembly (comparison with positional de Bruijn graph assembly) 20

Subgraph tracking 20

Performance optimization 21

## SUPPLEMENTAL RESULTS

### Simulated data

To determine the best-case accuracy and sensitivity of GRIDSS, and to compare performance to other methods, we developed an extensive set of simulated data. Our approach was to simulate a range of events on human hg19. For performance reasons and due the inability of some variant callers to detect inter-chromosomal events, only intra-chromosomal events were simulated. Chromosome 12 was chosen as it has close to the median chromosome size and GC content of the human genome, happens to be rich in oncogenes, and has been previously used for similar simulations. To allow false positive calls to be assigned based on event type, each simulation consists of events of a single type only. Six heterozygous variants sets were created: deletion, insertion, inversion and tandem duplication, and two translocation sets.

For deletion, insertion, inversion and tandem duplication event types, events of size 1, 2, 3, 4, 5, 6, 7, 8, 9, 10, 12, 16, 20, 24, 28, 32, 48, 64, 80, 96, 112, 128, 160, 192, 224, 256, 288, 320, 512, 1024, 2048, 4096, 8192, 16384, 32768, and 65536 base pairs (bp) were simulated for a total of 18,000 variants of each type. Variants were positioned such that each variant had at least 2,500bp separation from all ambiguous reference bases (Ns) and all other variants.

Translocation events were simulated by fragmentation of hg19 Chr 12 into 2500bp segments and concatenation of a subset in random orientation. Two rearrangement data sets were generated, one with rearrangement events in which the fragmentation was performed at random, and a second designed to contain more difficult to detect events. This second set was generated ensuring each rearrangement contained repetitive sequence on one side by selecting only 2,500bp sequences starting or ending at a SINE/Alu element as annotated in the UCSC hg19 RepeatMasker annotation. In both data sets, fragments with ambiguous reference bases were excluded from reassembly. In both data set, the variant chromatid contains 10,000 fragments.

For each of the 6 data sets, paired-end reads were generated at 100× coverage using the supplied Illumina MiSeq error profiles of ART 1.51 with 100bp read length and 300bp fragment size with a standard deviation of 10% of the mean fragment length. Reads were aligned to the full hg19 reference genome using bwa mem 0.7.10-r789 and coverage was downsampled to 60×, 40×, 15×, 8× and 4× using Picard tools.

Software used for comparison was selected favouring popular tools with a cross-section of calling methods. Comparisons were made using the most recently released versions as of October 2015. GRIDSS results were compared to BreakDancer 1.4.5 (breakdancer-max), CREST 0.0.1, CLEVER 2.0rc3, Cortex 1.0.5.21, DELLY 0.6.8, GASVPro 20140228, Hydra-Multi 0.5.2, LUMPY 0.2.11, Pindel 0.2.5b6, Socrates 1.1, Manta 0.29.6, and VariationHunter (CommonLawRelease 0.04 using mrfast 2.6.0.1).

All software was run according to the settings recommended by the user guide, software website, README, or publication in that order of preference. Conversion scripts to VCF version 4.1 were written for software using custom output formats with the VCF calling location set to the start of the confidence window and the CIEND field set to the end of the confidence window. Memory and CPU statistics were collected for each caller by invoking the POSIX time command against a wrapper bash shell script. Variants were categorised as true positives if all event breakpoint locations were within 100bp of the called interval and the called event size was within 25% of the actual event. As BreakDancer does not report event orientation, breakend orientation was not considered when determining a match for any caller. For the chromothripsis-like simulation data set, variants 50bp or smaller were filtered to remove artefacts caused by inexact sequence homology around the breakpoint positions. For performance reasons, callers other than GRIDSS were run only on the 2×100bp, 300bp fragment bwa data slice for a total of 439,540 simulated variants per caller.

Scripts used to perform the simulations and comparisons are located in the src/test/sim and src/test/r subdirectories of the GRIDSS source code.

A number of structural variant callers were excluded from analysis for the following reasons:

GASVPro: results were significantly worse than expected for a read pair-based caller. Upon investigation it was found that the LLR scoring used by GASV excessively favoured very large events. Combined with a filter removing overlapping events, most true positive GASV calls were filtered by the cluster pruning algorithm. For example, in NA12878 a false positive deletion call from chr1:32,060,879 to chr1:243,114,737 with a LLR of 1.4x10^10^ resulted in the removal of all chr1 deletion calls within this interval, thus removing most chr1 calls. Under these circumstances, it was decided that GASVPro results were not representative of read pair-based caller performance and results were excluded.

CLEVER: on simulated data, CLEVER called many fewer results than expected (41 deletion calls, 0 for other events). Through personal correspondence with the software author it was determined that all published version (1.1, 2.0rc1 and 2.0rc3) contained critical bugs causing either program failure, or call failure). As the recommended solution to compare against the most recent unstable, unreleased development version lacking both version and release information is not appropriate for reproducible evaluation, CLEVER results was excluded.

CREST: CREST failed to complete on 5 data sets. CPU usage indicated that CREST was running, but the output showed no progress was being made. For one data set, CREST was left to execute for 120 days without progress before being forcibly terminated. Although CREST could be run on the failing data sets if aligned with bowtie2, this may have confounded CREST results. Thus CREST was excluded.

laSV: laSV could not be run as the required auxiliary reference genome files were not present in the <http://hgdownload.soe.ucsc.edu/goldenPath/hg19> reference data set, and no documentation was provided regarding the extraction format for the custom RepeatMasker track used by laSV. Additionally, neither the pre-packaged reference nor the example test data for laSV were publically accessible as of 20 December 2015.

BreaKmer: not designed to scale for whole-genome analysis^[[1]](#footnote-1)^.

VariationHunter: No results for VariationHunter could be obtained. VariationHunter crashed with a “Segmentation fault (core dumped)” error on all simulations. VariationHunter could not be run on NA12878 in a timely manner due to requirement of in excess of 20,000 hours of computation time required to run mrfast on the 50× whole genome sequencing data set.

Comprehensive simulation and analysis of translocations

To further test GRIDSS, a comprehensive simulation across a range of read lengths, fragment sizes and depths of coverage was performed on GRIDSS using the chromothripsis-like simulation. Using the same protocol as the earlier simulation, paired-end reads were generated at 100× coverage using ART 1.51 with read lengths of 36, 50, 75, 100, 150 and 250bp and normally distributed fragment lengths of 150, 200, 250, 300, 400 and 500bp with standard deviation of 10% of the mean fragment length. Reads were aligned using novoalign 3.02.13 and bowtie2 2.2.5 as well as bwa mem 0.7.10-r789 and coverage down-sampled to 60×, 40×, 15×, 8× and 4×.

GRIDSS was run on all combinations of read length, read depth, fragment size and aligner except combinations in which the mean fragment size was less than or equal to the read length. A total of 13,831,272 variants were simulated. Performance for each is summarised by taking the harmonic mean of sensitivity and precision (F score). As can be seen from Supplemental Figure S3 and Supplemental Figure S4, reciprocal assembly support is more difficult to obtain both with shorter reads, and in the presence of repetitive sequence.

GRIDSS was robust to the choice of aligner across the entire input range with only a minor performance penalty for bowtie2 compared to alignment with bwa mem or novoalign. With a 500bp fragment size, the bowtie2 penalty increased significantly with the cause being traced to a known issue with the default settings of bowtie2 when aligning concordant fragments larger than 500bp^[[2]](#footnote-2)^.

Whilst unimportant for single nucleotide variant (SNV) calling, the library fragment length distribution plays an important role in SV detection with the effect most noticeable at lower levels of coverage. For 2×100bp reads, the sensitivity of GRIDSS at 8× with 500bp fragments significantly exceeds that at 15× with shorter 150bp fragments (90% vs 78%). This trend is present across all read length (36-250bp) and fragment sizes (150-500bp) (Supplemental Figure S4). In general, libraries with longer fragment lengths improve contig length as well as directly increasing the variant spanning read pair coverage hence the signal strength of true variants.

### Contig assembly rate

To determine the coverage required for assembly for a given read length and fragment size, assembly and supporting read counts were extracted for each breakpoint of true positive GRIDSS variant calls. As shown in Supplemental Figure S6, the GRIDSS assembly rate depends primarily on the read length, fragment size, and number of supporting reads. In all cases, increasing the number of supporting reads increases the likelihood of assembly. For shorter read lengths, there is an increased likelihood of both missing assembly as well as fragmented assembly (multiple assemblies for single breakend due to insufficient read overlap). In the extreme case of 2×36bp with 500bp fragments, assembly remains heavily fragmented even at high coverage. Even with such high levels of fragmentation, libraries with longer fragment sizes outperform shorter libraries due to the greater number of supporting reads for a given read depth. It should be noted that even if assembly is fragmented and a single contig cannot be assembled, variant calling is still improved as GRIDSS imposes no penalty for support from multiple assemblies and the assembly contigs can still be more accurately aligned than their shorter constituent reads.

### Subgraph Assembly

The same error correction and graph compression algorithms that are used in the positional de Bruijn graph implementation are used and contigs are called on the error corrected subgraph. The subgraph is divided based on the presence of reference support. For each connected subsubgraph of nodes with no reference support, the longest weighted path is calculated subject to the following constraints:

- The path must start at a successor to a node with reference if possible
- The path must end at a predecessor to a node with reference if possible
- The path must satisfy one of the above criteria if no paths satisfy both

The longest weighted path problem is a well-studied computer science problem which has proven to be NP-hard in the general case, but solvable in linear time for directed acyclic graphs and the standard directed acyclic graph algorithm is used for finding the best path with slight modification. The number of nodes traversed is recorded and if a safety threshold is reached, full path traversal is aborted and path finding falls back to a simple greedy traversal to prevent exponential runtime in degenerate edge cases such as occurs in highly repetitive genomic regions.

Once the best breakend path has been identified, the start and end of the path is extended using longest weighted path traversals restricted to only nodes providing reference support. An assembled contig is created from the underlying *k*-mers. The location and nature of the assembly is determined by the existence of starting/ending reference supporting nodes. How the assembly is treated is determined by whether breakend sequence is flanked on both, one, or neither side by reference supporting sequences:

- both: assembly supports an indel between the expected positions of the breakend flanking *k*-mers.
- one: assembly indicates a large structural variation from the expected position of the flanking *k*-mer to an undetermined location.
- neither: assembly indicates a large structural variation but the exact breakpoint location could not be determined. A breakpoint position confidence interval is calculated from the contributing read pairs.

### Assembly comparison results

GRIDSS was run using both assembly algorithms on the 50× NA12878 Platinum Genomics data set and assembly rates compared over a range of *k*-mer sizes (10, 13, 16, 19, 22, 25, 28, and 31). Since GRIDSS encodes each sequence *k*-mer within a 64 bit integer using the 2 bit UCSC packedDna encoding scheme (<https://genome.ucsc.edu/FAQ/FAQformat.html#format7>), the maximum *k*-mer size supported by GRIDSS is a 32mer thus the range of *k*-mers compared is representative of the *k*-mer sizes available to GRIDSS.

Hg19 RepeatMasker annotations were downloaded from UCSC and the assembly rates per top-level repeat class were calculated. Each assembly was assigned to a single repeat class based on the repeat class with the greatest overlap of the assembly position. Assemblies not overlapping any repeat annotated were classified as background. As can be seen in Supplemental Figure S7, the background assembly rate outside of repetitive sequence is lower in positional assembly compared to windowed assembly. Reflecting the robustness with respect *k*-mer size of positional assembly variant calling, the raw assembly rates of positional assembly is robust to choice of *k*-mer size. Windowed assembly lacks this robustness and assembly rates increase at a greater than exponential rate with decreasing *k*-mer size.

Common across both assembly approaches in the increase in the raw assembly rate in regions of repetitive sequence compared to the unannotated background rate. Whilst most repeat classes observe an assembly rate approximate 4-fold higher than the background rate, low complexity, simple repeats, SINE elements and “other” repeats have a 20 fold increased rate of assembly. Due to the repetitive nature of these elements, this increased rate is not unexpected and is only problematic if the resultant assemblies have a high rate of high quality false positives. Supplemental Figure S8 shows that these regions are indeed enriched for high scoring assemblies, but this enrichment is restricted to windowed assembly only.

GRIDSS assemblies can be anchored on one side, both sides, or neither side, representing a rearrangement to a remote location, an assembly spanning a small indel, or an assembly with an imprecise breakpoint respectively. Supplemental Figure S9 shows that by breaking down assembly rates according to the assembly anchoring, a high rate of high quality assemblies with flanking anchors is present in windowed assembly, but not in positional assembly.

Supplemental Figure S10 breaks down the flanking anchors assembly further by looking at the assembly type both before and after realignment. For assemblies anchored on only one side, the assembly rate for both positional and windowed assembly are similar, indicating a similar detection capability for large rearrangement events. For intra-window small events, the windowed assembly detection signal is drowned out by the false positive noise caused by sequence homology. For a pre-realignment assembly to have flanking anchors in positional de Bruijn graph assembly, a putative SV read with sequence homology must exist in the expected anchoring position, but with windowed de Bruijn graph assembly, such a read can exist anywhere within the graph assembly window and thus the resultant higher rate of flanking anchors. As a result, the windowed assembly approach as used in GRIDSS is not appropriate for intra-window event calling.

### Performance in repetitive regions

GRIDSS optionally supports the analysis of read alignments with multiple reported mapping positions (described below). However, applications such as precision cancer medicine require high specificity and focus on interpretable or actionable calls, so by default GRIDSS does not consider multi-mapping reads. What separates GRIDSS from callers that do not handle multi-mapping reads is that GRIDSS is able to partially recover multi-mapping read support through assembly without the ten-fold computational and storage overhead of multi-mapping alignment. Since GRIDSS considers multi-mapping reads as unmapped, multi-mapping reads fall into one of two categories: lost reads consisting of reads from read pairs for which both reads are unmapped or multi-mapping, and recoverable reads from read pairs for which one read is uniquely mappable. These recoverable reads are treated as OEAs and, with sufficient coverage, can be assembled into a single contig. Since these contigs are both longer than the read length and have sequencing errors corrected, they are more easily mappable than individual reads. Many repetitive regions that are not uniquely mappable at the read level are uniquely mappable at the contig level.

Using this process of indirect recovery of multi-mapping reads, GRIDSS is able to maintain a low false discovery rate in repeats other than low complexity, simple, and satellite repeats (Supplemental Figure S11). RepeatMasker annotations of the lung cancer data indicate that 37% of the high-confidence GRIDSS calls were SVA or LINE translocations.

This approach allows GRIDSS to identity breakpoints even when one breakend consists of entirely multi-mapping reads. On the 778 neochromosome data, GRIDSS was able to correctly identify a breakpoint located in a SINE element that was support by zero uniquely mappable split reads or read pairs. The 318bp breakpoint contig assembled by GRIDSS was uniquely aligned to the correct SINE element (exact alignment match, next closest differed by 6bp) even though every constituent reads was incorrectly aligned.

When compared to multi-mapping aware callers, the GRIDSS approach has both advantages and disadvantages. Since GRIDSS relies on uniquely mapping anchoring reads, GRIDSS is unable to call variants callable by multi-mapping aware callers in which both sides of a breakpoint are multi-mapping at the read level. Conversely, whilst multi-mapping aware callers have a limited number of mapping locations they consider (e.g. VariationHunter defaults to 300 positions), GRIDSS has no such limitation.

### Performance on complex somatic rearrangements

To test the performance of GRIDSS on complex genomic rearrangements, we used it to detect rearrangements in published cancer-associated neochromosome datasets [[17](#_ENREF_17)]. Neochromosomes arise following chromothripsis and multiple circular breakage-fusion-bridge cycles, and contain hundreds of rearrangements. The sequencing data was derived from flow-isolated neochromosomes from well/de-differentiated liposarcoma cell lines: 778 (RRID:CVCL_M808), GOT3 (RRID:CVCL_M819), and T1000 (RRID:CVCL_M809). The reads were primarily 100nt paired end reads, although the 778 cell line data comprised 35, 75 and 100nt long reads. Reads were aligned with bowtie2. GRIDSS was run with default parameters on each of the three samples. Variants outside the published neochromosome contiguous genomic regions (CGRs) were filtered. GRIDSS calls were compared to the published calls, which were predicted using a bespoke discordant read pair method and had undergone extensive manual curation. Calls were considered equivalent if both breakpoints were within 200bp. Events of less than 500bp were filtered as per the methods used in the published call set.

Across the all three samples, GRIDSS showed strong agreement with the published rearrangements with a sensitivity of 98% (1,335/1,361), with only 2.6% supported by only one-sided or no breakpoint assemblies (31, 4 rearrangements respectively). By refining the call set to base pair accuracy, GRIDSS was able to identify breakpoint microhomology in 64% of rearrangements, untemplated inserted sequence in a further 10%, with the remaining 26% of breakpoints characterised by blunt end-joining.

Upon investigation of a number of breakpoints in which GRIDSS and the published call set disagreed concerning the rearrangement partner, a number of compound rearrangement events were identified. GRIDSS was able to identify compound rearrangements involving an additional short fragment from a third genomic location omitted in 5% (64) of the published rearrangements as well as a identifying a further 66 compound rearrangements in which the spanning fragment could not be unambiguously placed. The compound rearrangements were identified by GRIDSS assembly contigs spanning not only the rearrangement event for which the assembly was generated, but also an additional nearby rearrangement. Since the published call set is based on supporting read pair, these compound rearrangements containing short fragment were misclassified as simple rearrangements, as a sufficient number of read pairs spanned the intervening short fragment.

The value of performing variant calling after assembly is highlighted by the rearrangements identified by GRIDSS but not detected by the original discordant read method in the published rearrangement set. The increased sensitivity of GRIDSS allowed 551 additional rearrangements to be identified of which 364 either fell below the 7 read pair threshold used in the construction of the published call set, or lay within 1000bp of another rearrangement. Of these, 233 rearrangements were supported by reciprocal breakpoint assemblies and a further 112 by single-sided assembly.

### Comparison to other callers on neochromosome call set

To compare GRIDSS to the other callers on the neochromosome call set, all callers were run on a subset of the input. As not all callers are capable of handling multiple libraries containing different read lengths, callers were compared using only the 2×100bp libraries. Callers using aligned reads as input were run against both bwa mem and bowtie2 aligned reads with the result with the highest sensitivity used. Calls were matched if both breakends were within 180bp, the event sizes differed by no more than 25%. Events of less than 500bp were filtered as per the methods used in the published call set. Unmatched calls outside of the published Contiguous Genomic Regions (CGRs) were ignored. Results were aggregated across the 3 data sets. GRIDSS identified the most calls in the published call set, with up to 74% overlap between other callers, and the GRIDSS High Confidence call set (Supplemental Table S2).

### Validation on lung cancer

Lung tumours were collected from two stage II lung cancer patients (LP1-43 and LP2-33) who underwent surgical resection at the Austin Hospital, Melbourne, Australia. Tumour biopsies were taken for snap freezing in liquid nitrogen and for fixation in 10% buffered formalin. A pre-operative and a series of post-operative blood samples were collected from the participating patients. Plasma was separated from whole blood within 3 hours since its collection using two centrifugation steps, 800 and 1600 rcf for 10 minutes each. Buffy coat was also collected to use as a germline DNA control. All collected plasmas and buffy coats were then stored at -80°C until DNA extraction. Informed consent was obtained from the two patients and the study was approved by the Institutional Review Board of Human Research Ethics Committee.

Primary lung cancer tumours were whole genome sequenced to 40× coverage on an Illumina HiSeq generating paired 100bp reads. Reads were aligned using bowtie2 version 2.2.5 with --qc-filter -X 1000 --local command line parameters and duplicates marked using Picard tools. Variants were sorted by quality score reported by GRIDSS and the following filters applied:

- Variants not in the GRIDSS high confidence call set were filtered.
- dGV (Database of Genomic Variants) hits within 10bp were filtered.
- Alternate contig variants were filtered.
- Variants less than 1Mb were filtered.
- Translocation under 5kbp were filtered.
- Variants without split read, read pair, and assembly support were filtered.
- Telomeric and centromeric variants were filtered.
- Variants with a NCBI blast hit (all human sequences) spanning the breakpoint were filtered.

Candidate rearrangements were manually inspected using IGV and 15 highest scoring variants with a change in copy number or SNV allele frequency supporting somatic origin were selected as candidates for validation. Of these 15 candidates, 8 were selected for validation (4 from each patient) based on the ease of PCR primer design.

Genomic DNA was extracted from fresh frozen and formalin-fixed tumours, and buffy coat using the DNeasy Blood and Tissue Kit (Qiagen, Hilden, Germany). Plasma DNA was extracted from 2 mL of plasma using the QIAamp Circulating Nucleic Acid kit (Qiagen) by eluting twice with 50 uL of ACE buffer.

Primers were designed to flank the rearrangement breakpoints and to generate less than 100 bp amplicons for fragmented formalin-fixed and plasma DNA. The validation experiments were carried out on a CFX Connect (BioRad). PCR reaction mix in a final volume of 20 uL contained; 1× PCR buffer, 2.5 mM MgCl2, 100 nM each primer, 200 µM of dNTPs, 5 µM of SYTO 9, 0.5 U of HotStar Taq DNA polymerase (Qiagen) and 10 ng of genomic DNA. The cycling and melting conditions were as follows; one cycle of 95ºC for 15 min; 55 cycles of 95ºC for 10 s, 68-60ºC (gradient PCR) for 20 s and 72ºC for 30 s, followed by one cycle of 97ºC for 1 min and a melt from 70ºC to 95ºC temperature rising 0.2ºC per second.

The presence of tumour DNA containing the validated genomic rearrangements was tested in a series of plasma DNA samples using the QX200 droplet digital PCR system (BioRad). PCR mix was prepared with EvaGreen ddPCR supermix, 100 nM each primer and 2 uL of plasma DNA in a final volume of 23 uL. The manufacturer’s instruction was used for droplet generation, PCR cycling and droplet reading. Analysis of the ddPCR data was performed with QuantaSoft analysis software (Bio-Rad).

### HCC1395 cell line genomic breakpoints identification

HCC1395 tumour DNA sequences were downloaded for SRP022140 from sra.dnanexus.com. Of the nominal 63× coverage, effective coverage was only 21× as SRR892402, SRR892256, and SRR866931 contain identical sequences, as do SRR866925, SRR892454, and SRR892651.

Reads were aligned using bwa mem with default parameters. GRIDSS 0.10.4 was run with default parameters. GRIDSS calls were matched to the 240 junctions (including the 102 missing validation probes), and to all 26 genomic breakpoints with 250kbp and 1kbp margin respectively using https://github.com/PapenfussLab/sv_benchmark/blob/master/R/HCC1395.R. Matching calls were then manually filtered to exclude variants with position or orientation not consistent with the gene fusion (Supplemental Table 3). Putatively causative variants were manually filtered by inspection in IGV. Of the 3 genomic breakpoints not identified by GRIDSS, zero split read or read pairs supporting *SHISA5*/*RP11-24C3.2* could be found, and *NOM1*/*MNX1-AS2* and *CCDC117*/*CTA-292E10.6* were each supported by only a single read pair able to be explained as the sequencing of a long fragment. In addition, GRIDSS was able to find 3 additional causative genomic breakpoints not previously identified. Concerningly, neither *NOM1*/*MNX1-AS2*, *CCDC117*/*CTA-292E10* nor *MAVS*/*PANK2*, *WARS*/*DEGS2*, and *RP11-85K15.2*/*BAZ1A* are able to explain the either corresponding fusion transcript. In all these cases, read-through gene fusion not only is still required to explain the transcript, but the transcript can be can explained purely though read-through gene fusion without the presence of any genomic breakpoint.

### Variant scoring model comparison

In addition to the empirical reference likelihood model used as the GRIDSS default, we investigated three additional models (Supplemental Figure S13). All models result in similar results with the empirical reference likelihood models performing 4% better that read counts when performance is defined as the area under the curve truncated at 1000 false positives.

#### Read Count model

This model weights all reads equally. As each read is given a score of 1, this matches the read counting used by other calls for ranking variants.

#### MAPQ model

This model ignores the empirical library distributions and weights according to the Phred-scaled likelihood of the read being correctly mapped.

#### Empirical log-likelihood ratio model

Under this model, we compare the likelihood of a structural variant $V$ between genomic locations $a$ and $b$ of variant allele frequency $\alpha$ that best matches the data, and the null reference allele hypothesis $R$. Under the null (reference allele) hypothesis, no structural variations exist and all mapping errors are assumed to be uniformly distributed noise. Variants are scored according to the log likelihood ratio (LLR) of the variant and the null reference models. In general, the probably of evidence $e$ given allele $A$ is of the form

$$\Pr\left( e | A \right)=\Pr\left( M \right)\cdot\Pr\left( e | M\wedge A \right)+\Pr\left( \overline{M} \right)\cdot\Pr\left( e | \overline{M}\wedge A \right)$$

Where $M$ is a correct mapping of the evidence, and $\Pr\left( e | M\wedge A \right)$ and $\Pr\left( e | \overline{M}\wedge A \right)$ are determined by the empirical distribution of the evidence. When the mapping is incorrect, the read is uninformative with $\Pr\left( e | \overline{M}\wedge A \right)=\Pr\left( e | \overline{M}\wedge R \right)$. The empirical distributions of read and read pair mappings for each library are calculated so that probability distributions $P_{sc}\left( l \right)$ of a mapped read having a soft clip of length $l$, $P_{rp}\left( f \right)$ of a read pair mapping with inferred fragment length $f$, and the probabilities $p_{d}$ of read pair mapping discordant, and $p_{u}$ of a single read of the pair unmapped are known.

For split reads $sr$, $\Pr\left( M \right)=q_{a}q_{b}$ and $\Pr\left( e | M\wedge R \right)=P_{sc}\left( l_{a} \right)$, where $l$ is the length of the of soft clip and $q_{i}$ is the correct read mapping probability reported by the aligner at $i$.

$$\log\left( \frac{\Pr\left( sr | V \right)}{\Pr\left( sr | R \right)} \right)=log\left( \frac{\Pr\left( M | V \right)\cdot\Pr\left( sr | M\wedge V \right)+\Pr\left( \overline{M} | V \right)\cdot\Pr\left( sr | \overline{M}\wedge V \right)}{\Pr\left( M | R \right)\cdot\Pr\left( sr | M\wedge R \right)+\Pr\left( \overline{M} | R \right)\cdot\Pr\left( sr | \overline{M}\wedge R \right)} \right)$$

$$=log\left( \frac{q_{a}q_{b}\cdot P_{sc}\left( 0 \right)+\left( 1-q_{a}q_{b} \right)\cdot P_{sc}\left( l_{a} \right)}{q_{a}q_{b}\cdot P_{sc}\left( l_{a} \right)+\left( 1-q_{a}q_{b} \right)\cdot P_{sc}\left( l_{a} \right)} \right)$$

$$=log\left( \frac{q_{a}q_{b}\cdot P_{sc}\left( 0 \right)+\left( 1-q_{a}q_{b} \right)\cdot P_{sc}\left( l_{a} \right)}{P_{sc}\left( l_{a} \right)} \right)$$

$$=log\left( q_{a}q_{b}\frac{P_{sc}\left( 0 \right)}{P_{sc}\left( l_{a} \right)}+\left( 1-q_{a}q_{b} \right) \right)$$

Similarly, discordant read pairs $dp$ are modelled as $(q_{a}, q_{b},f)$ with $\Pr\left( M \right)=q_{a}q_{b}$ and $\Pr\left( e | M\wedge A \right)=p_{d}+P_{rp}\left( f_{A} \right)$.

$$\log\left( \frac{\Pr\left( dp | V \right)}{\Pr\left( dp | R \right)} \right)=log\left( q_{a}q_{b}\frac{p_{d}+P_{dp}\left( f_{V} \right)}{p_{d}+P_{dp}\left( f_{R} \right)}+\left( 1-q_{a}q_{b} \right) \right)$$

### Runtime Performance

Runtime performance of GRIDSS was tested using 50× coverage whole genome human sequencing on an isolated virtual machine (dedicated dual-socket Xeon E5-2690v3, hyperthreading enabled, 512GB physical memory, 20 cores/492GB assigned to ESXi 6.0.0 CentOS release 6.4 VM, 2×8Gb Fibre Channel connections to a shared 3PAR SAN enclosure containing 480 900GB 10,000 rpm SAS HDDs). GRIDSS was configured to use 20 threads (notably not all SV methods are multi-threaded). Timings are indicative of expected performance, but may vary depending on the setup. GRIDSS consumed 381 minutes of total CPU time and took 236 minutes wall clock time. Of the 236 minutes wall clock time, 159 minutes was spent on file I/O and parsing of the input over three separate passes (library metrics calculation, evidence extraction, reference coverage annotation), with only 38, 11, and 11 minutes spent on multi-threaded stages of assembly, soft clip alignment by external aligner, and variant calling respectively. These times could be further optimised within an analysis pipeline. GRIDSS assembly memory consumption is less than 1Gb per assembly thread with usage dominated by the data structures associated with read *k*-mer contribution tracking.

By breaking down runtime performance of GRIDSS by processing stage, bottlenecks and multithreading effectiveness can be ascertained. Both external alignment and assembly scale with multiple threads and, in a multithreaded environment, execution time is dominated by single-threaded processes that traverse the raw input file (Supplemental Figure S14). Since assembly multithreading is performed at the reference chromosome level, the maximum theoretical improvement is limited to the maximum chromosomal assembly runtime, for NA12878 50× WGS, the limiting chromosomes are 1 and 2 taking 12 and 15 minutes respectively, with all other chromosomes completing in under 9 minutes.

### Assembly Memory Consumption Breakdown

Since the assembly window is extended to ensure the highest scoring contig can have no supporting reads overlapping with any partially constructed contig, the assembly memory construction is theoretically bounded by only the size of the input. This theoretical upper bound can be realised through the creation of an input in which each putative assembly contig is followed by a nearby putative assembly contig with greater support. In practice, memory consumption is much lower, as outlined in Supplemental Figure S15. The assembly graph contained an average of only 62.7 reads when calling a putative forward contigs on Chr 12 of the NA12878 platinum genomics 50× 2×100bp data set. The majority of these contigs were supported by singletons with only 42,855 of the 730,000 putative contigs called passing basic filtering (at least 3 reads support, not aligning to reference). With an average of 3770 nodes, GRIDSS assembly memory consumption is dominated by the *trackerLookup* hash table which records which positional de Bruijn nodes are supported by which reads.

Whilst most data structures contain relatively few records, peak size for all structures is at least two orders of magnitude larger than the mean. These peaks generally occur in repetitive regions of the genome, with the largest peaks in centromeric regions and nuclear mitochondrial DNA (NUMTs) with corresponding increases in runtime. In extreme cases (such as >100,000× coverage of some centromeric sequences in 50× WGS data sets), a high-coverage 1kbp window will take longer to assembly than an entire chromosome. Such bottlenecks are mitigated through the blacklisting of the extreme high-coverage regions, and by the downsampling performed at discordant coverage extrema.

Due to improvements in the hg38 reference genome over hg19, utilising data sets aligned against hg38 instead of hg19 obviates the need to utilise the ENCODE DAC blacklist to remove problematic regions. On typical 40x WGS data, GRIDSS only throttles 82kb of hg38 sequence, compared to 15kb in additional to 11Mb of sequence in the DAC blacklist.

## SUPPLEMENTAL METHODS

### Read filtering

Due to the prevalence of artefacts in sequencing data, GRIDSS applies a number of filters designed to improve specificity by removing reads likely to interfere with variant calling. These are generally optional, but by default, the following read filters are applied:

- MAPQ of at least 10. The read mapping quality score (MAPQ) is defined by the SAM specifications as $-10\log_{10} (Pr(incorrect mapping))$. A low mapping quality score indicates that the read is unlikely to be mapped correctly. All reads with a MAPQ score of less than 10 are treated as unmapped. For split reads, both the read MAPQ and the soft clip re-alignment MAPQ must exceed the threshold.
- Maximum coverage of 10,000x. Regions of extremely high coverage may be due to a collapsed reference sequence in highly repetitive regions. Coverage of canonical sequences such as those present in the centromeres can be greater than 3 orders of magnitude higher than the nominal coverage and as such, regions with coverage greater than 10,000× are excluded from variant calling and assembly.
- Soft clipped reads must have at least 95% of aligned bases matching the reference sequence as these partially aligned, poorly matching reads are frequently alignment artefacts.
- Soft clipped reads must contain at least 4 soft clipped bases. Sequencing errors in the bases near either end of a read will result in Smith-Waterman local alignment of the read excluding the end bases from the alignment.
- Estimated Shannon entropy of aligned nucleotides (based on frequencies of nucleotides in the read) must be at least 0.5 bits. Reads with low complexity in the mapped bases (such as poly-A runs) were found to have a high false positive rate. If the estimated entropy of nucleotide frequency distribution within the read is less than 0.5 bits, the read is considered unmapped. For example, a read with sequence AAAAAAAAAGTTT with the final three bases soft clipped would have estimated entropy of $-\sum_{i\in\left\{ A,C,G,T \right\}} P\left( b_{i} \right)\log_{2} \left( P\left( b_{i} \right) \right)=-\frac{9}{10}\log_{2} \left( \frac{9}{10} \right)-0-\frac{1}{10}\log_{2} \left( \frac{1}{10} \right)-0\approx0.47$ bits and would be considered unmapped.
- Mean Phred-scaled base quality score of soft clipped bases must be at least 5. Soft clips containing runs of low base quality scores are filtered as such sequences are typically due to sequencing errors.
- Adapter filtering. Reads containing the first 12 bases of the Illumina Universal Adapter, Illumina Small RNA Adapter, or Nextera Transposase sequences are filtered if the sequence is homologous to the reference sequence at the aligned location.
- Overlapping read pairs from short fragments are excluded. A read pair aligned in the expected orientation with overlapping read alignments is indicative of a pair sequenced from a fragment shorter the combined read lengths. Such read pairs do not provide support for structural variants and are filtered. If soft clipped, each read is still individually considered for split read analysis and assembly. In the case that the fragment length is shorter than the read length, the reads may contain soft clips ‘dovetailing’ in adapter sequence past the other read. If there exists a micro-homology between the adapter and reference adjacent to the alignment position, the reads will be over-aligned and such read pairs will appear to be in an unexpected orientation. Such edge cases are filtered by allowing a 4bp dovetail when determining whether a read originates from a short fragment.

### Coverage blacklist

Alignment of whole genome sequencing data to the reference results in extreme coverage in some genomic regions, for example in satellite repeats. These regions are likely to contain false positives and are computationally expensive to assemble due to the high coverage. To mitigate the effect of such regions on variant calling and runtime performance, GRIDSS performs two independent operations. Firstly, GRIDSS filters all reads within 1 fragment length of any region of extreme coverage (default 10,000x). Secondly, GRIDSS down-samples before assembly in poorly mapped regions, where the density of variant-supporting reads is unusually high. When the number of SV-supporting reads within a sliding window (default 1,000bp) exceeds the threshold (default 5,000), only a subset of the reads are used for assembly. The probability of a SV-supporting read being retained is given by

$$Pr\left( \mathrm{retain} \right)=\left\{ \begin{aligned} 1, &size\leq a \\ e^{-\frac{size-a}{m}}, &x\geq0 \end{aligned} \right.$$

where $m$ is the target maximum number of reads in the window, and $a$ is the unconditional acceptable threshold in which no downsampling occurs (default 50% of maximum). This hybrid exponential back-off model ensures that no down-sampling is performed except for coverage outliers, and within coverage outliers, real events are unlikely to be filtered.

### Breakpoint position error margin

Split reads originating from the same event should only support a single breakpoint position. In practice, however, sequencing errors, imperfect homologies in the neighbourhood of the breakpoint, and alignment artefacts can result in variation of the breakpoint position supported by each read. As a result, split reads originating from the same event can provide support for breakpoints at different positions. To ensure such evidence results in a single call, an additional margin is added when determining clique assignment. By default, this margin is 10bp for variants 20bp or larger, linearly decreasing to no margin for 1bp thus ensuring that small non-overlapping indels are not merged.

### Windowed de Bruijn graph assembly (comparison with positional de Bruijn graph assembly)

To compare the effect of choice of assembly algorithm, a windowed assembler using a traditional de Bruijn graph was also implemented in GRIDSS. Windowed de Bruijn graph assembly is performed using a traditional de Bruin graph with a dynamically-sized sliding window. The sliding window is sized such that all supporting reads for a correctly assembled breakpoint contigs will be present in the de Bruijn graph when the associated contig is called. Reads supporting putative structural variants are added to the graph in-order in a single pass over the coordinate sorted intermediate files.

An initially empty annotated de Bruijn graph is constructed to which SV-supporting reads are added and removed. Graph edges are determined by *k*-mer adjacency, with each node in the graph containing the following attributes:

- *k*-mer: sequence of k (default k=25) bases.
- weight: *k*-mers are weighted by the minimum of the phred-scale base quality score of all bases spanned by the *k*-mer. Total *k*-mer weight is the sum of the weight for each contributing read *k*-mer.
- reference support: a *k*-mer supports the reference if all bases contained within that *k*-mer are locally aligned to the reference in at least one split read.
- expected positions: for split reads, the expected position is the starting position of the *k*-mer if the split read were fully aligned; for read pairs, the expected position is the position of the *k*-mer if the mate read were to be perfectly concordantly aligned with the read pair from a fragment of median size.

All read bases of split reads and the discordant pair mate reads are split into successive *k*-mers and added to an annotated de Bruijn graph in the order of their alignment position. Since the strand for the read is known from the alignment, only one of the sequence or reverse complement is added.

### Subgraph tracking

Since only reads supporting putative breakpoints are included in the graph, the de Bruijn graph consists of multiple isolated subgraphs, each supporting a different putative structural variant. Exploiting this property, the minimum and maximum expected position for each connected subgraph is tracked. When the current genomic traversal position reaches the point where subsequent reads will not support the same breakpoint as reads in a given subgraph (that is, the current position is more than 2 maximum concordant fragment lengths after the subgraph maximum expected position), subgraph assembly is performed, and the entire subgraph removed.

To prevent an unbounded graph size in highly repetitive regions, if the width of a subgraph exceeds a safety threshold (16 times maximum concordant fragment length), subgraph assembly is performed immediately.

### Performance optimization

#### Record caching

To assemble a contig supporting a putative structural variant at a given genomic location, the full read sequence of all supporting reads is required. By sorting the input and intermediate alignment files according to alignment position, retrieving one half of a DP or SR can be done efficiently as they are stored contiguously within the file. To improve the retrieval speed of the other half of a DP or SR, an additional copy is made and sorted by the genomic coordinate of the partner. Simultaneous traversal of both files enables both halves of a DP or SR to be accessed at both mapping locations with purely sequential file access. Actual disk storage required for all intermediate file varies considerably between data sets but is typically 5-25% of the size of input files.

#### Record streaming

For compatibility with existing tools and file formats, and to allow visualisation of intermediate results, reads are stored using the BAM file format. The SAM/BAM format defines the coordinate sort order position of a mapped read as the position of the first aligned base of the read. The order in which evidence is processed in GRIDSS is by the starting position of the 95% confidence interval of putative breakpoint positions. For split reads this is the position of the mapped base adjacent to the split, which only matches the coordinate sort order if the soft clip occurs at the start of the read. Similarly, depending on the strand to which the local read is aligned, the start of the breakend interval for read pairs occurs either *maximum expected fragment size – mapped read length* bases before the first mapped base, or after the final mapped base.

The distance between the breakend start position and the position of the first base is bounded. For soft clips, it is bounded by the maximum mapped read length, and for read pairs by the maximum expected fragment size and the maximum mapped read length. This allows evidence to be reordered in-memory by buffering all reads within *max(maximum expected fragment size, maximum mapped read length)* of the current position. GRIDSS implements this buffer as a priority queue sorted by breakend start position.

#### Blacklisting

Since reads with low mapping quality are filtered before assembly, multi-mapping reads in repetitive genomic regions do not affect assembly performance. Other sequences such as satellite, centromeric and telomeric repeats have a considerable effect on assembly performance. These are regions that may be poorly assembled in the reference or unrepresentative of individuals. Alignments to these regions are likely dominated by reads from paralogous sequences not present in the reference. Without any explicitly blacklisted regions, single-threaded assembly of NA12878 50× WGS takes 504 minutes which is reduced to 145 minutes if regions in the ENCODE DAC blacklist are ignored.

#### Assembly graph size contraints

Although the locally maximal contig for any given read is the globally maximal contig, the number of nodes loaded before a maximal path is reads is unbounded. For any given read, a nearby successor read could form part of a higher scoring contig, which in turn could have a higher scoring nearby successor. As coverage increases, the likelihood of these successors existing also increases. When tested on 500× coverage WGS data, these chains were up to 10Mb in length.

Whist the priority queue of loaded contigs sorted by score enables maximal contigs to be identified, it does not ensure that the earliest maximal contig is at the head of the queue when the graph is mutated due the contig deletion. Take the scenario of contigs A1, B2, C3 and D2, with the AB, BC and CD pairing close by. Calling A requires B to be loaded, which requires C to be loaded which will load D as part of the graph traversal. If the maximal C is called which results in the removal of C and B from the graph, A1 and D2 will remain. Using a priority queue, A will not be assembled until D is called, even if A and D cannot contain the same reads. For high-coverage data, this results large number of low-scoring contigs remaining in the graph for extended periods of time. To constrain memory consumption, contigs more than 10 times the maximum expected fragment size from the frontier are called whenever the width of the graph exceeds 100 times the maximum expected fragment.

1. https://github.com/ccgd-profile/BreaKmer/issues/5 [↑](#footnote-ref-1)
2. http://sourceforge.net/p/bowtie-bio/bugs/323/ [↑](#footnote-ref-2)
