# Supplementary figures and images for: GRIDSS: sensitive and specific genomic rearrangement detection using positional de Bruijn graph assembly
Source: Genome Res. 2017 Dec;27(12):2050–60. doi: 10.1101/gr.222109.117 (PMC5741059; doi:10.1101/gr.222109.117)

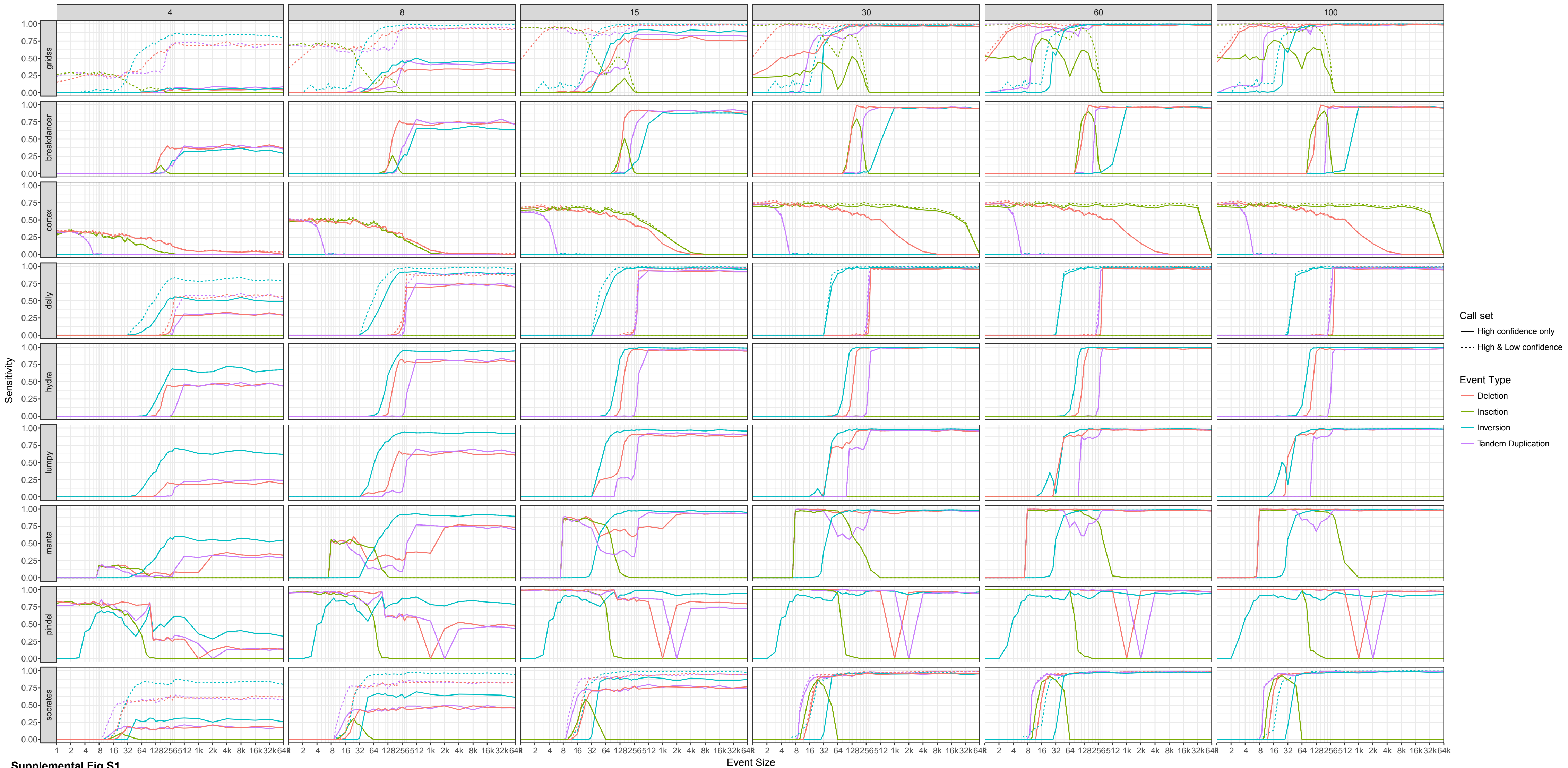

Supplement: Supplemental Material [file supp_gr.222109.117_Supplemental_Fig_S1.pdf]

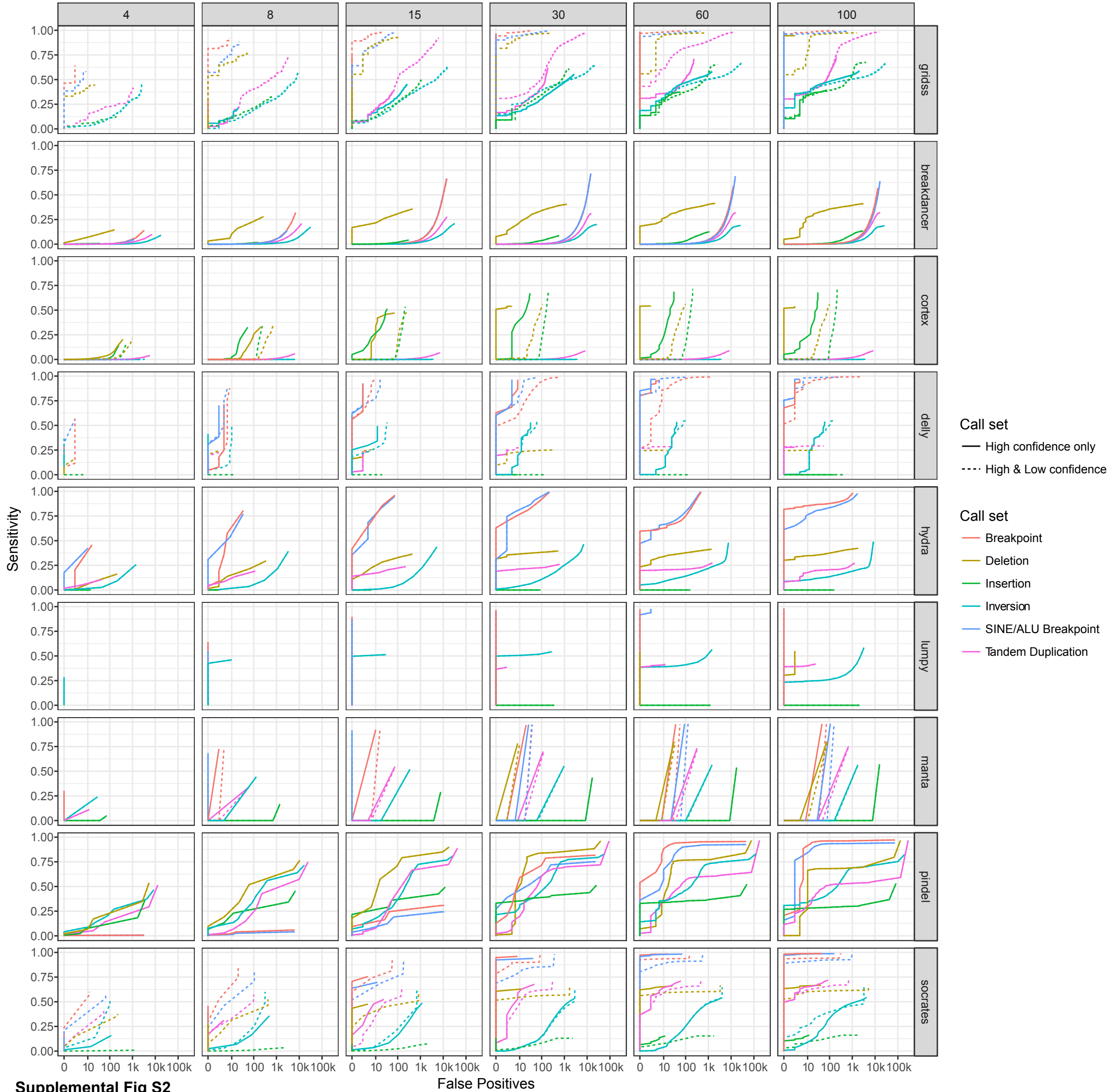

Supplement: Supplemental Material [file supp_gr.222109.117_Supplemental_Fig_S2.pdf]

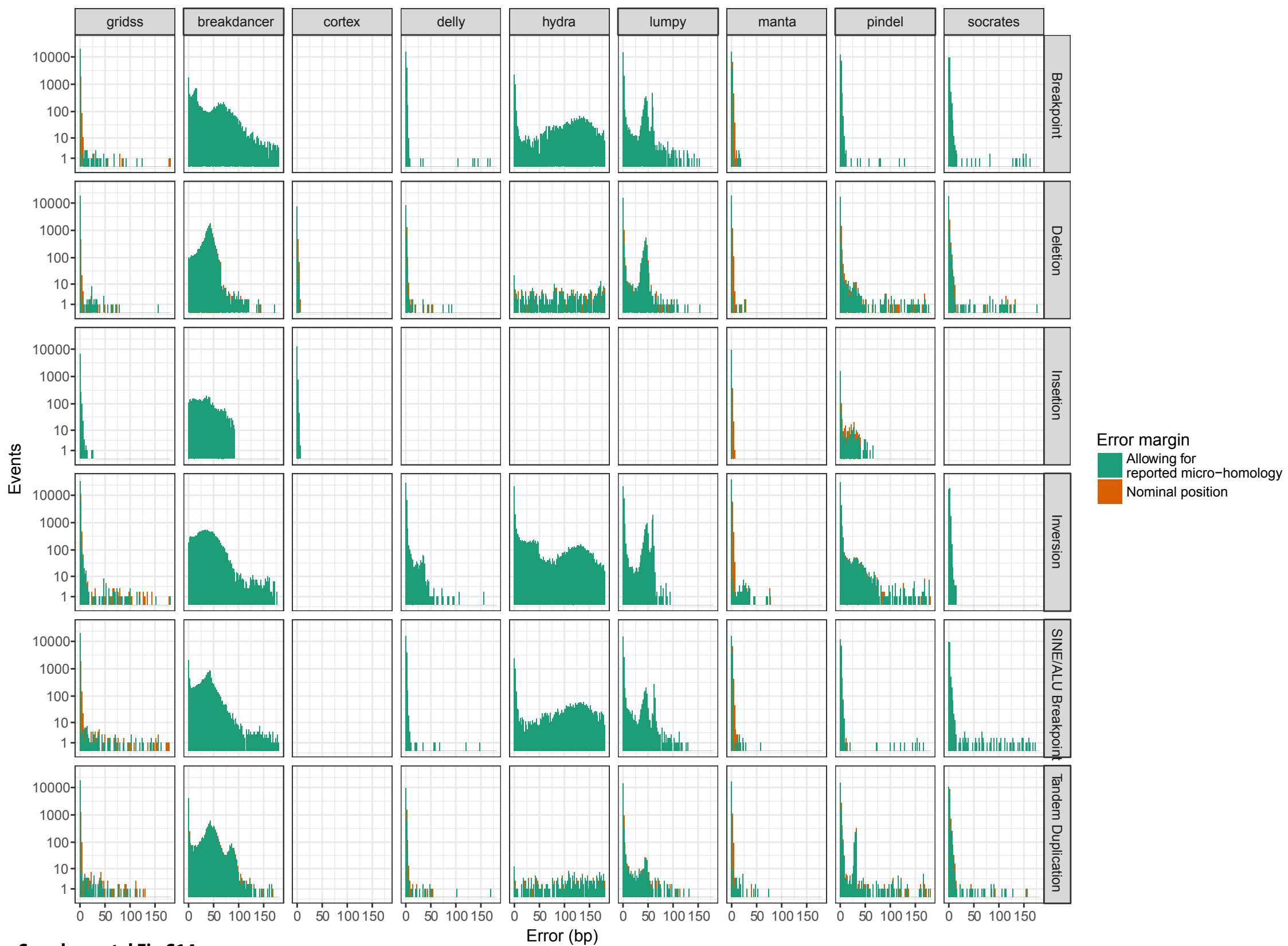

Supplement: Supplemental Material [file supp_gr.222109.117_Supplemental_Fig_S14.pdf]
