# Supplementary material for: GRIDSS: sensitive and specific genomic rearrangement detection using positional de Bruijn graph assembly
Source: Genome Res. 2017 Dec;27(12):2050–60. doi: 10.1101/gr.222109.117 (PMC5741059; doi:10.1101/gr.222109.117)
Supplement: Supplemental Material [file supp_gr.222109.117_Supplemental_Fig_S4.pdf]

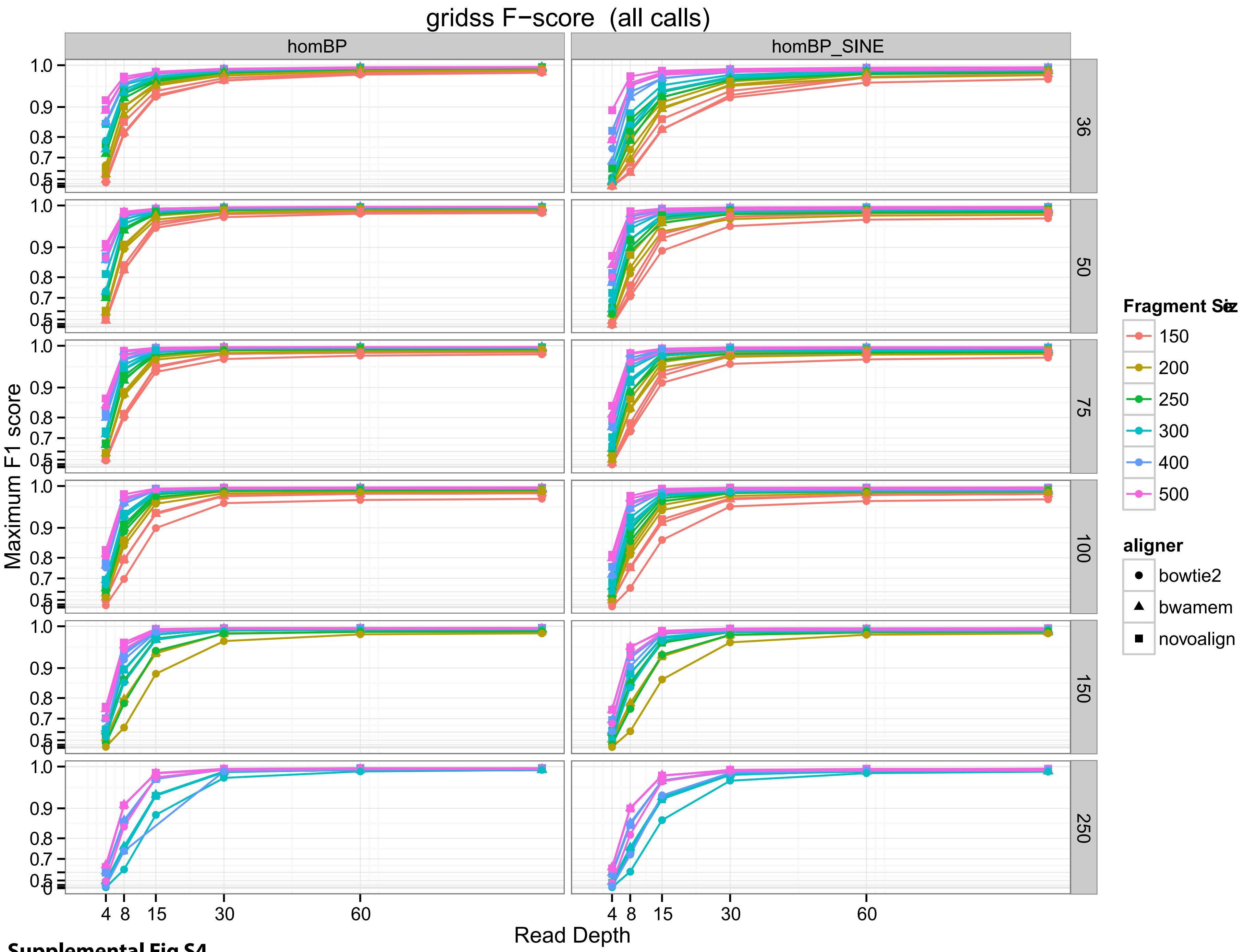

Supplemental Fig S4

F-scores for GRIDSS calls with reciprocal assembly support. At 30x allelic coverage, GRIDSS can detect rearrangement events under most conditions. Performance varies with fragment size and read length with best performance on 75-100bp with long fragment sizes. Relative to the other aligners, bwa mem performs poorly with 36bp reads whilst bowtie2 has worse performance at 75bp or longer. Non-linear scales have been used.
