# Supplementary material for: GRIDSS: sensitive and specific genomic rearrangement detection using positional de Bruijn graph assembly
Source: Genome Res. 2017 Dec;27(12):2050–60. doi: 10.1101/gr.222109.117 (PMC5741059; doi:10.1101/gr.222109.117)
Supplement: Supplemental Material [file supp_gr.222109.117_Supplemental_Fig_S5.pdf]

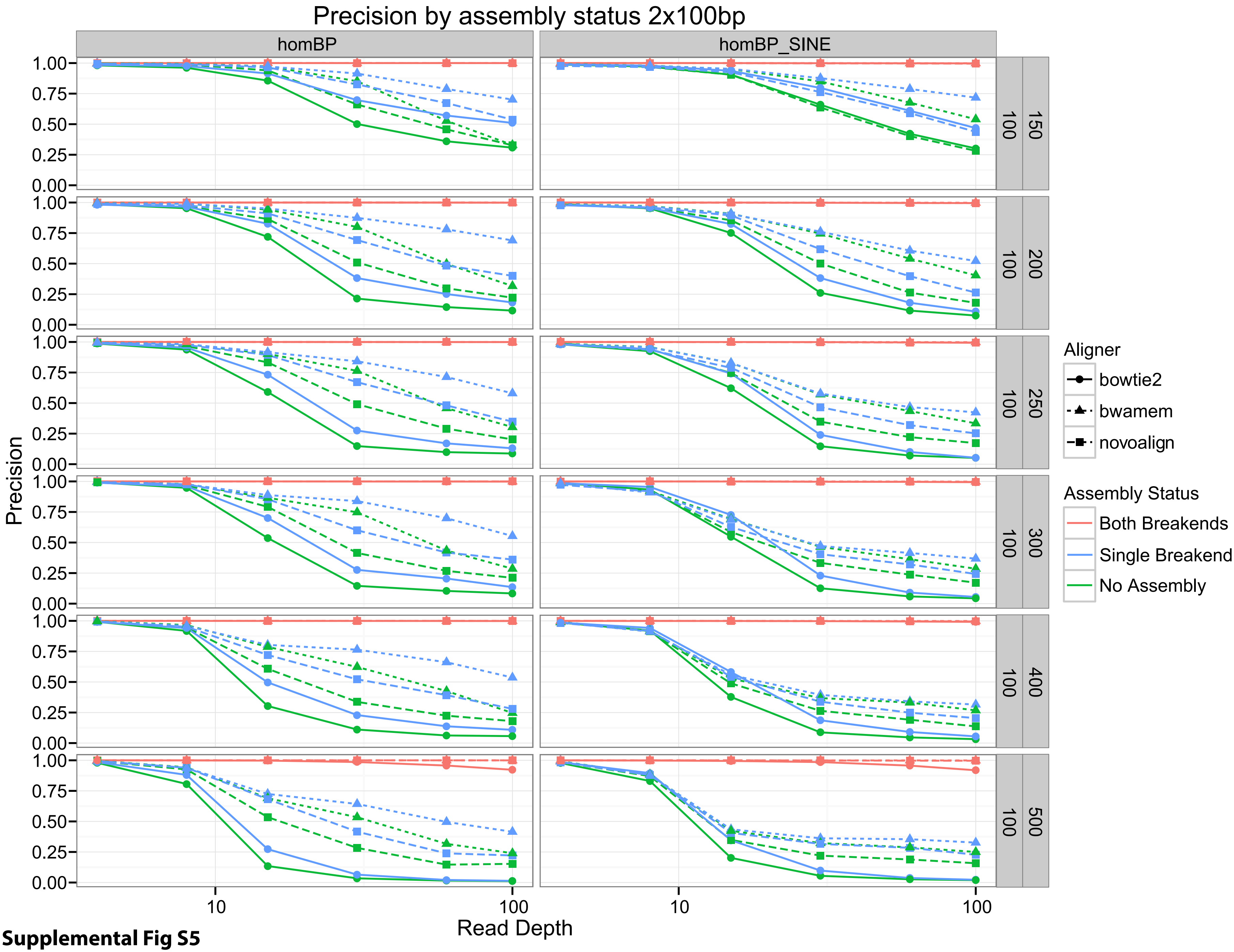

Supplemental Fig S5

GRIDSS precision. At low coverage, assembly support at both breakpoint is not present. From 15x allelic coverage onwards, precision of calls supported by only one-sided assembly or with no assembly support drops dramatically, whilst precision of calls with reciprocal assembly support remains close to 100% under all conditions.
