# Supplementary material for: GRIDSS: sensitive and specific genomic rearrangement detection using positional de Bruijn graph assembly
Source: Genome Res. 2017 Dec;27(12):2050–60. doi: 10.1101/gr.222109.117 (PMC5741059; doi:10.1101/gr.222109.117)
Supplement: Supplemental Material [file supp_gr.222109.117_Supplemental_Fig_S6.pdf]

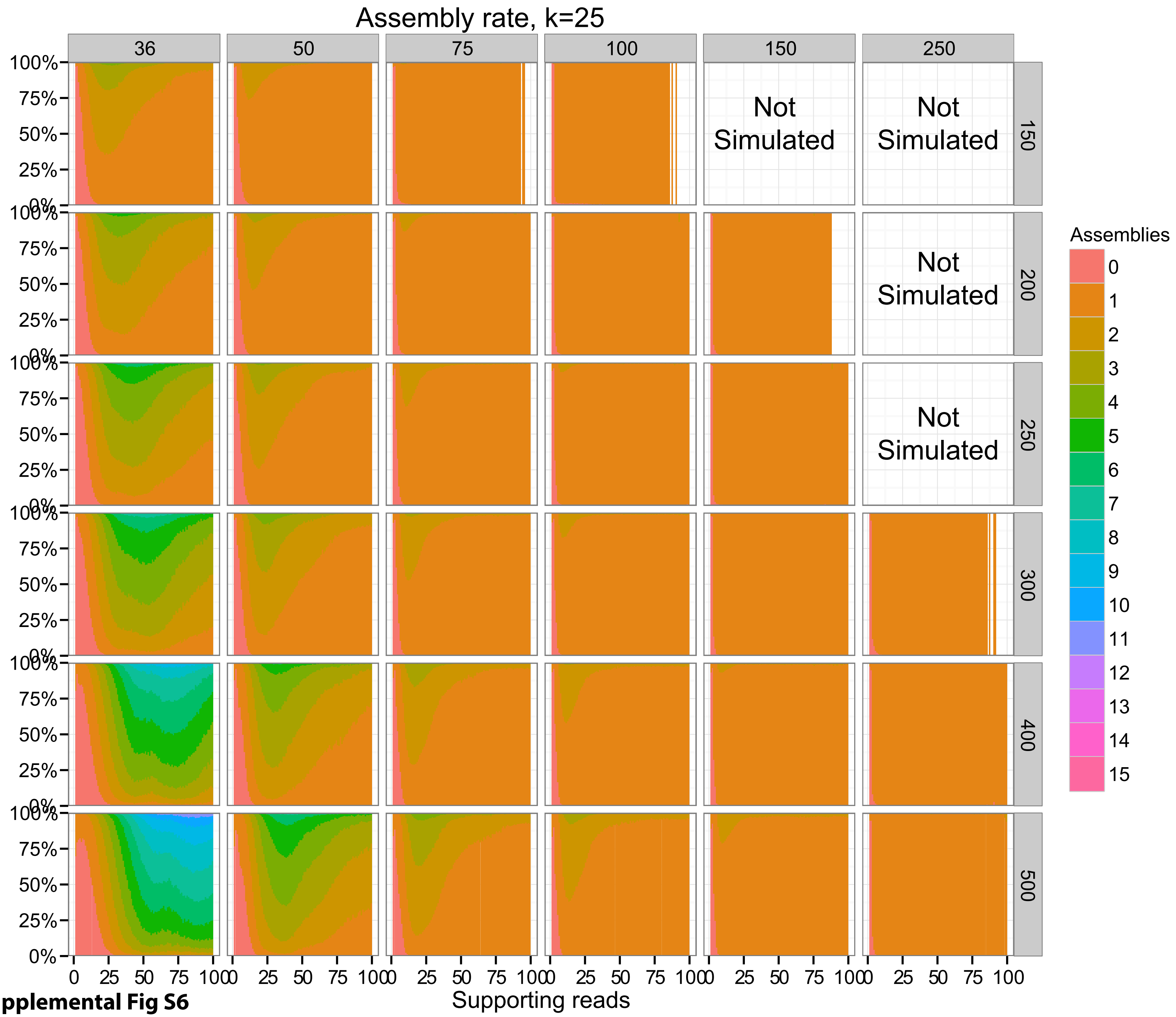

Supplemental Fig S6

Breakdown of number of supporting assemblies generated by number of reads supporting the breakpoint. For 100bp onwards, assemblies can be reliably generated from the minimum of 3 supporting reads required for GRIDSS assembly. For shorter read lengths, larger fragment sizes result in fragmented assembly with multiple assemblies mutually supporting the breakpoint. Note that for a given read depth, the expected number of supporting reads increases linearly with fragment size.
