# Supplementary material for: GRIDSS: sensitive and specific genomic rearrangement detection using positional de Bruijn graph assembly
Source: Genome Res. 2017 Dec;27(12):2050–60. doi: 10.1101/gr.222109.117 (PMC5741059; doi:10.1101/gr.222109.117)
Supplement: Supplemental Material [file supp_gr.222109.117_Supplemental_Fig_S7.pdf]

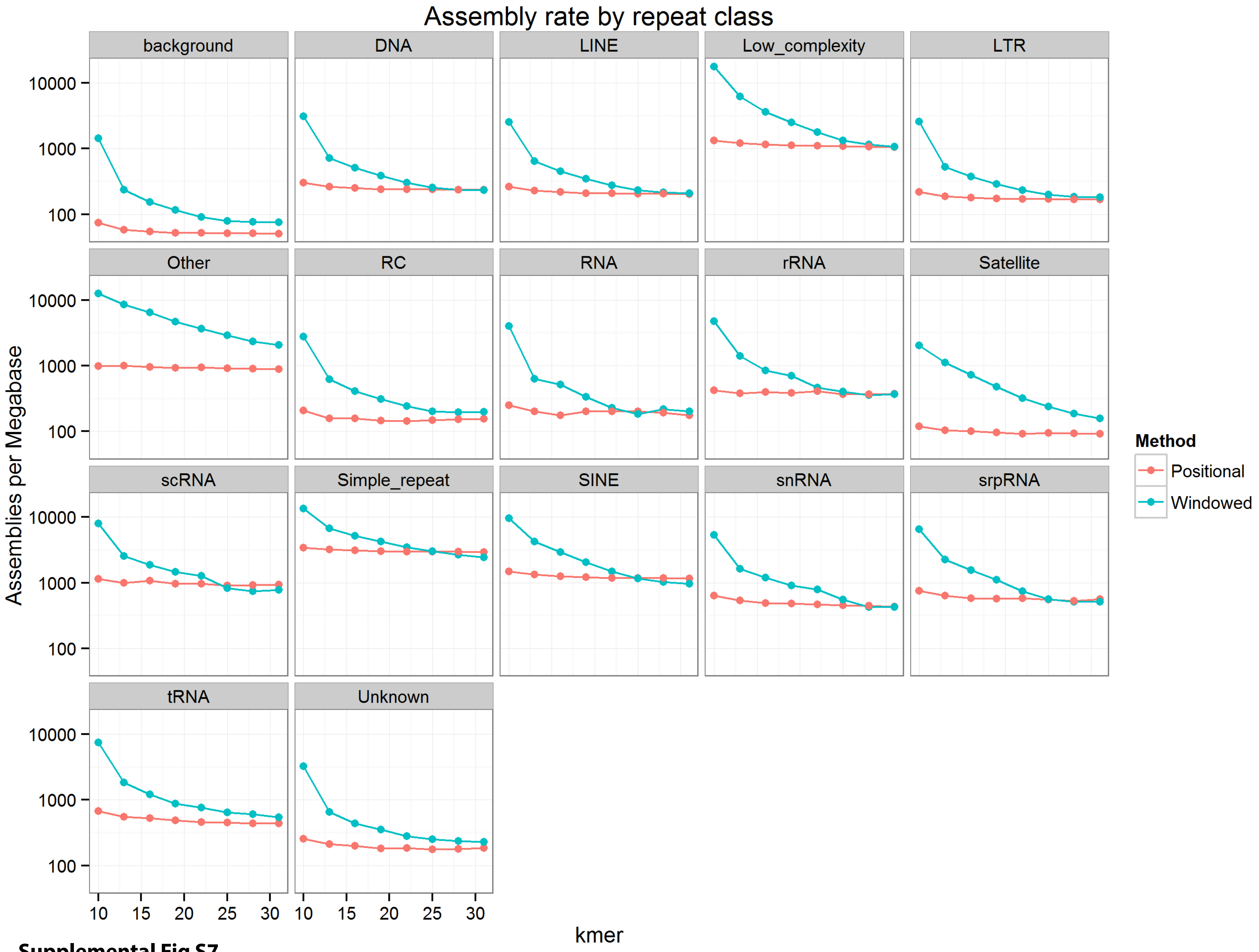

Supplemental Fig S7

Breakdown of assembly rates by RepeatMasker top-level repeat class annotations. Unannotated regions are labelled as background. Positional de Bruijn graph assembly allows for more robust assembly using short k-mers
