# Supplementary material for: GRIDSS: sensitive and specific genomic rearrangement detection using positional de Bruijn graph assembly
Source: Genome Res. 2017 Dec;27(12):2050–60. doi: 10.1101/gr.222109.117 (PMC5741059; doi:10.1101/gr.222109.117)
Supplement: Supplemental Material [file supp_gr.222109.117_Supplemental_Fig_S8.pdf]

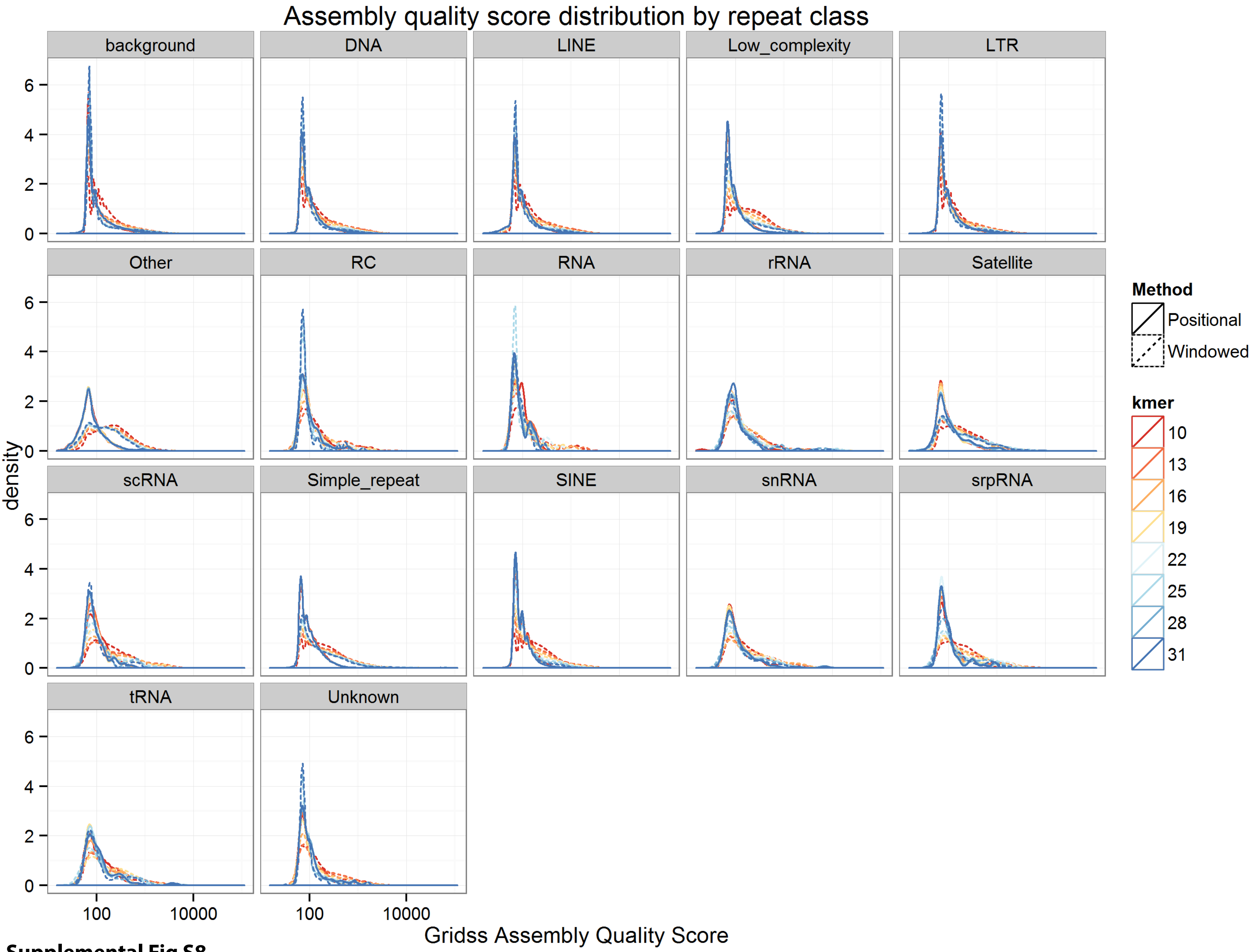

Supplemental Fig S8

Assembly quality score distribution by repeat type. Over-assembly of low complexity and simple repeats regions results in high confidence false positive calls using windowed assembly, but not positional assembly.
