# Supplementary material for: GRIDSS: sensitive and specific genomic rearrangement detection using positional de Bruijn graph assembly
Source: Genome Res. 2017 Dec;27(12):2050–60. doi: 10.1101/gr.222109.117 (PMC5741059; doi:10.1101/gr.222109.117)
Supplement: Supplemental Material [file supp_gr.222109.117_Supplemental_Fig_S9.pdf]

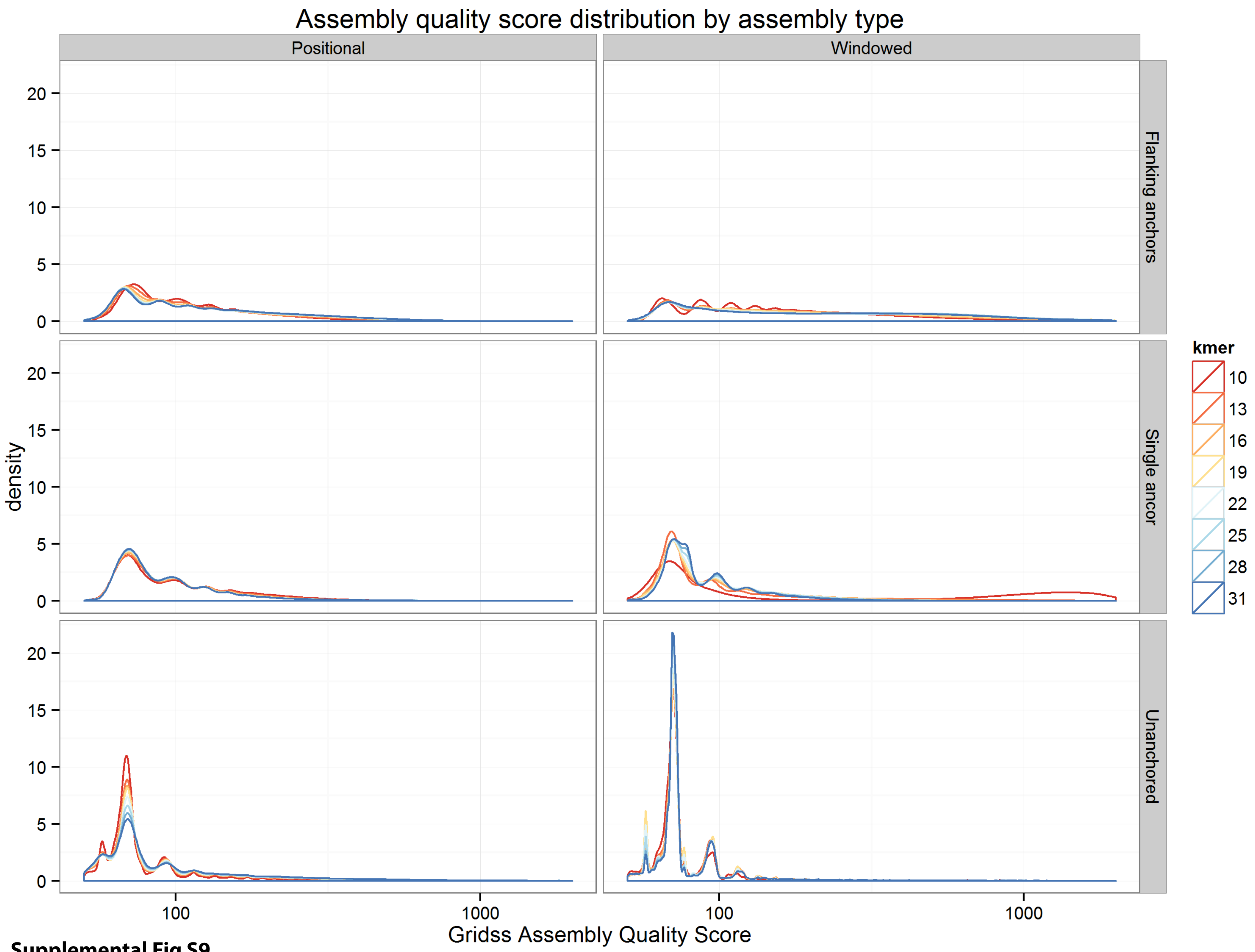

Supplemental Fig S9
Distribution of assembly scores by assembly method and anchor type. A high rate of flanking anchor assembly using windowed assembly is indicative of intra-window misassembly due to sequence homology. This misassembly does not occur when using positional de Bruijn graph assembly.
