# Supplementary material for: GRIDSS: sensitive and specific genomic rearrangement detection using positional de Bruijn graph assembly
Source: Genome Res. 2017 Dec;27(12):2050–60. doi: 10.1101/gr.222109.117 (PMC5741059; doi:10.1101/gr.222109.117)
Supplement: Supplemental Material [file supp_gr.222109.117_Supplemental_Fig_S10.pdf]

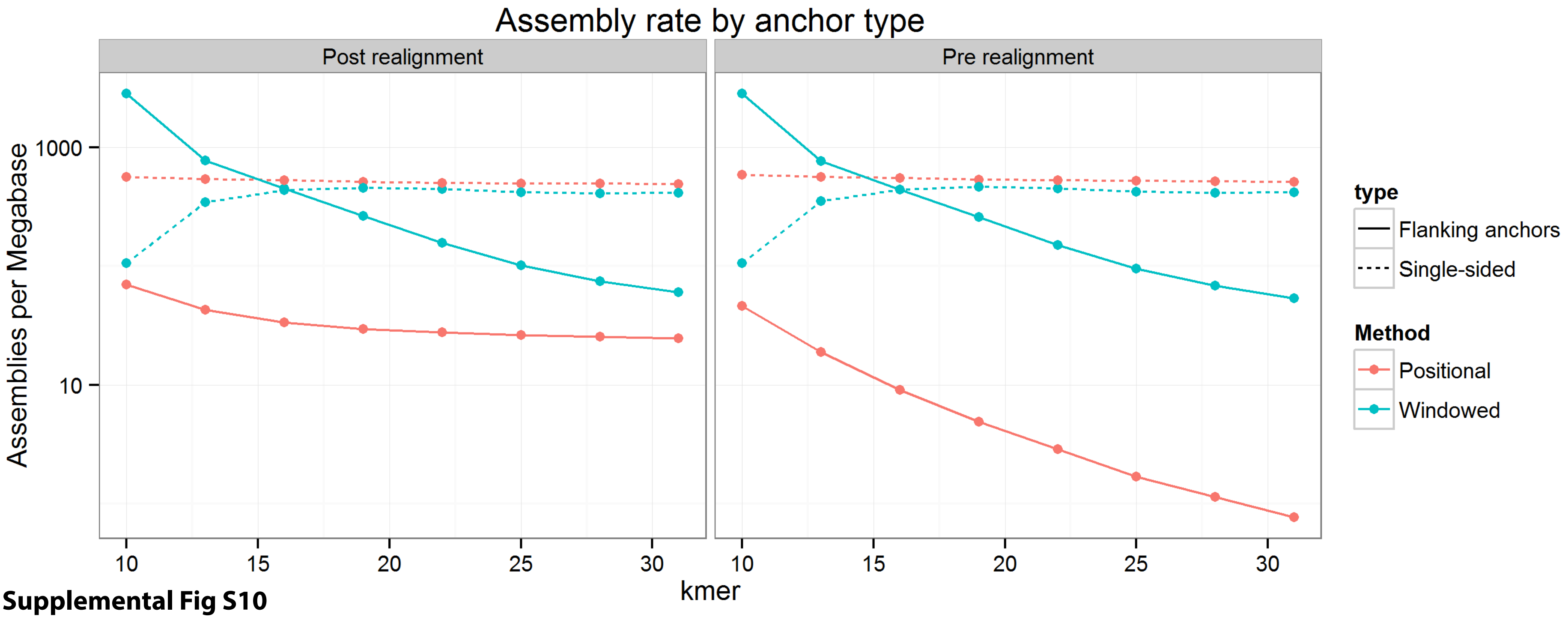

Supplemental Fig S10

Assembly rate by anchor type. Whilst both windowed and positional assembly has similar detection capability for large events in which only a single side of the event can be assembled, windowed assembly calls orders of magnitude more intra-window events due to misassembly of unrelated reads containing sequence homology. The trend of a reducing post realignment flanking anchor assembly indicates that a k-mer size of 40-50 would be required for windowed assembly performance to match that of positional assembly with k-mer of 20 for intra-window events.
