# Supplementary material for: GRIDSS: sensitive and specific genomic rearrangement detection using positional de Bruijn graph assembly
Source: Genome Res. 2017 Dec;27(12):2050–60. doi: 10.1101/gr.222109.117 (PMC5741059; doi:10.1101/gr.222109.117)
Supplement: Supplemental Material [file supp_gr.222109.117_Supplemental_Fig_S11.pdf]

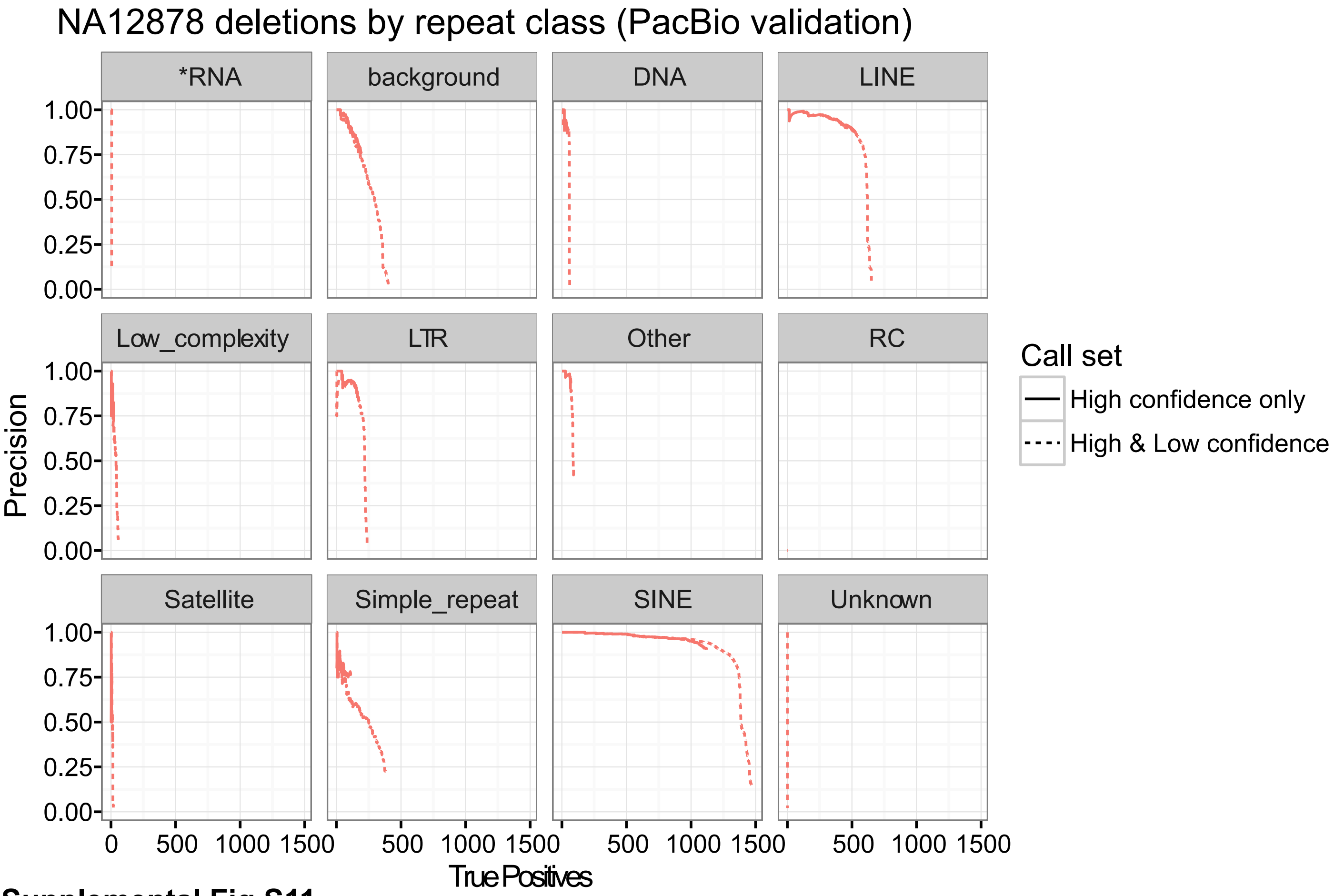

Supplemental Fig S11

Variant calls by repeat class. Breakpoints are broken down by RepeatMasker repeat class annotation. RNA repeat classes have been merged, background indicates no repeat annotation. GRIDSS is able to robustly call structural variants in large repeats but has a higher false discovery rate in low complexity, simple, and satellite repeats.
