# Supplementary material for: GRIDSS: sensitive and specific genomic rearrangement detection using positional de Bruijn graph assembly
Source: Genome Res. 2017 Dec;27(12):2050–60. doi: 10.1101/gr.222109.117 (PMC5741059; doi:10.1101/gr.222109.117)
Supplement: Supplemental Material [file supp_gr.222109.117_Supplemental_Fig_S12.pdf]

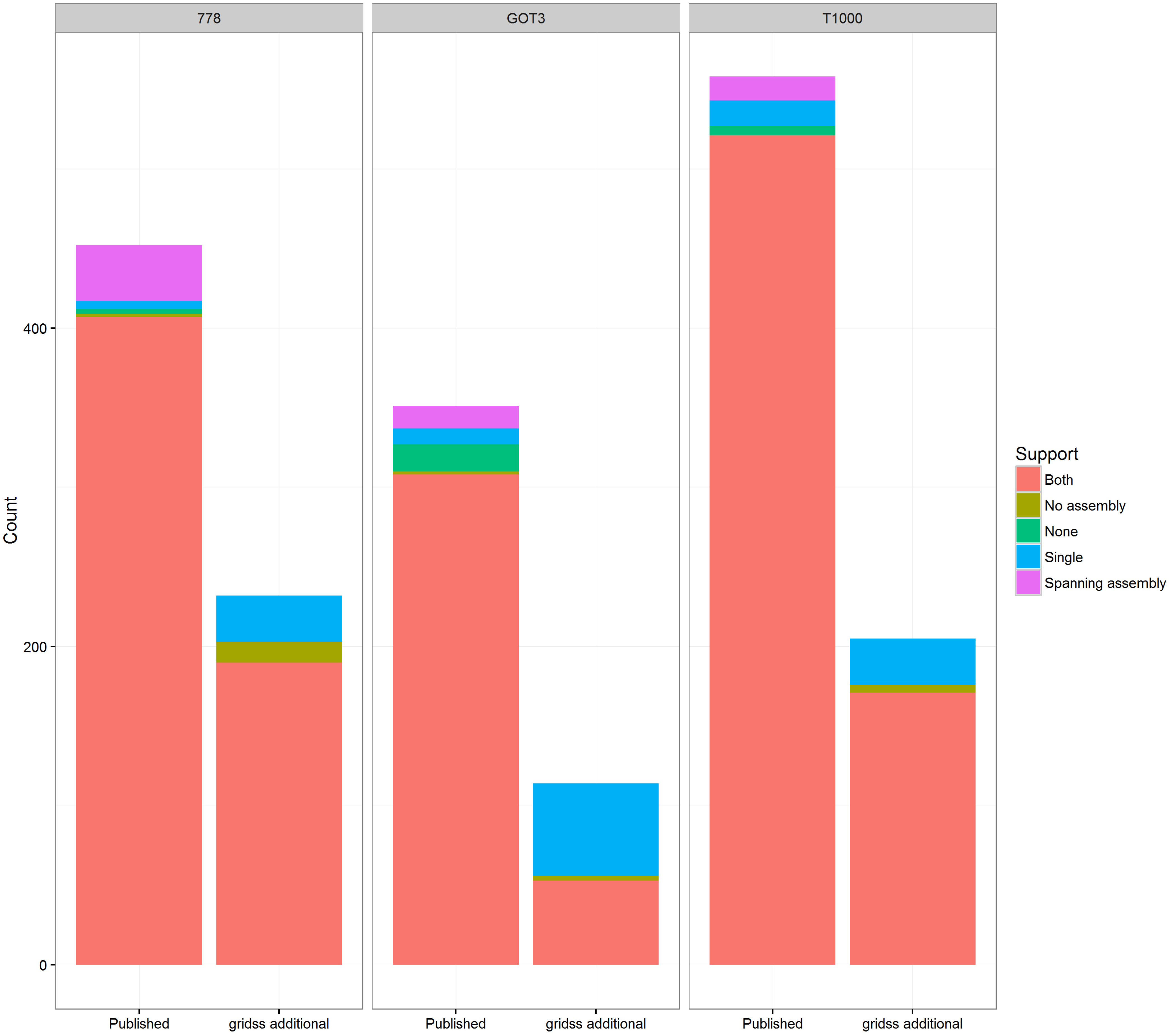

Supplemental Fig S12

Neochromosome comparison with published call set. GRIDSS identified 61 compound rearrangements misclassified by the published call set. 78% of the additional calls made by GRIDSS fell below the 7 read pair support threshold used by the published call set or lay within 1000bp of another rearrangement.
