# Supplementary material for: GRIDSS: sensitive and specific genomic rearrangement detection using positional de Bruijn graph assembly
Source: Genome Res. 2017 Dec;27(12):2050–60. doi: 10.1101/gr.222109.117 (PMC5741059; doi:10.1101/gr.222109.117)
Supplement: Supplemental Material [file supp_gr.222109.117_Supplemental_Fig_S13.pdf]

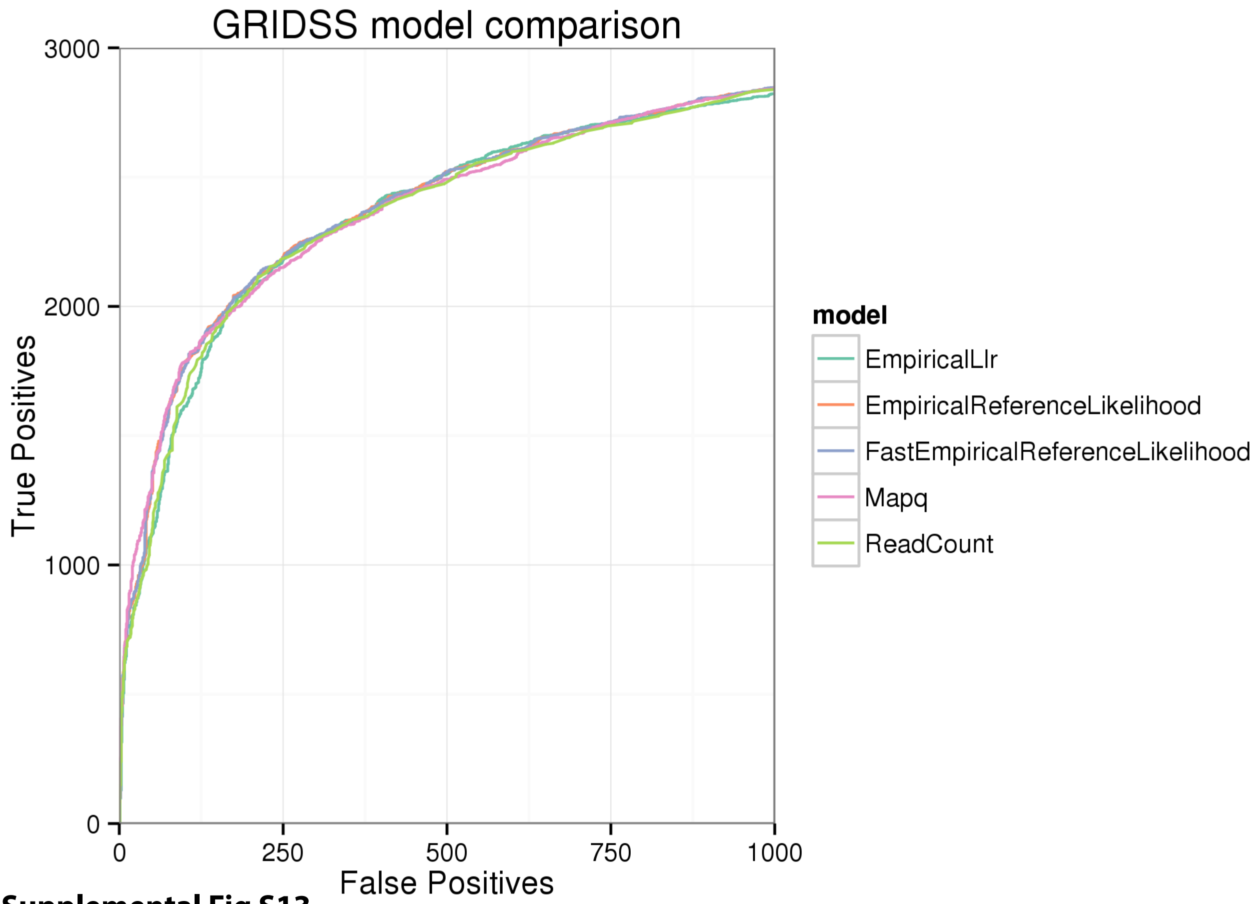

Supplemental Fig S13

ROC curve for alternative GRIDSS models for platinum genomics 2x100bp 50x NA12878 using Moleculo/PacBio long read truth set. The probabilistic variant scoring makes a small contribution to GRIDSS performance.
